# Residue-Specific Modulation of Aggregation-Associated Interactions by Spermine in Tau, α‑Synuclein, and Aβ40

**DOI:** 10.1021/jacsau.6c00126

**Published:** 2026-03-12

**Authors:** Debasis Saha, Xun Sun, Wangfei Yang, Jinghui Luo, Wenwei Zheng

**Affiliations:** † College of Integrative Sciences and Arts, 52448 Arizona State University, Mesa, Arizona 85212, United States; ‡ Center for Life Sciences, 28498 Paul Scherrer Institute, Forschungsstrasse 111, Villigen PSI 5232, Switzerland; § Center for Biological Physics, Arizona State University, Tempe, Arizona 85281, United States

**Keywords:** intrinsically disordered protein, protein aggregation, spermine, liquid−liquid phase separation, all-atom molecular dynamics

## Abstract

Preventing neurodegenerative diseases associated with intrinsically disordered proteins (IDPs) remains a major challenge due to the lack of a detailed, sequence-level picture of disease-relevant structure formation and the influence of cellular factors that modulate these transitions. Here, we probe spermine (Spm), a +4 charged polyamine abundant in cells, to determine how it reshapes the conformational ensembles and fibril-associated contact propensities of three disease-linked IDPs: the K18 domain of Tau, α-synuclein (αS), and amyloid-β40 (Aβ40). Using long all-atom molecular dynamics simulations across a range of Spm concentrations, we quantify residue-level changes in intrachain contacts relative to native contacts observed in fibrils and corroborate computational predictions with ThT fluorescence assays for Tau constructs. Spm acts in a sequence- and region-specific manner, not simply through the overall net charge. In K18, Spm binds near the fourth microtubule-binding repeat, disrupting intrachain contacts associated with Alzheimer’s fibril structures and thereby inhibiting aggregation. In αS, Spm binds mainly to acidic residues in the C-terminal half of the sequence and redistributes intramolecular contacts to enhance aggregation-prone interactions in the central region, providing a residue-level mechanistic basis for previously reported Spm-enhanced αS aggregation. For Aβ40, Spm neutralizes acidic residues near positions 22–24 and shifts intrachain interactions toward its aggregation-prone core, resulting in a net promotion of fibril-like conformations. These divergent effects show that net charge alone cannot predict the polyamine influence on IDPs. Instead, residue-specific binding hotspots and local reweighting of aggregation-linked contacts determine whether Spm promotes or suppresses fibril-like conformations. This combined simulation–experimental framework provides a mechanistic basis for how small molecules reprogram IDP conformational ensembles and suggests principles for designing ligands that exploit similar residue-level modulation.

## Graphical abstract

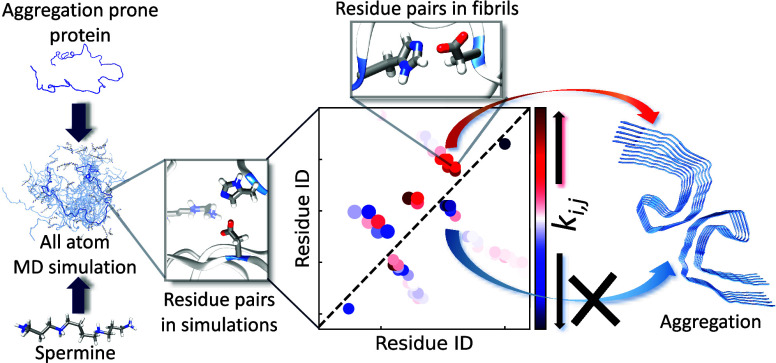

## Introduction

The rising prevalence of age-related neurodegenerative diseases among the elderly represents a persistent socioeconomic challenge. These conditions are often marked by the intracellular or extracellular aggregation of various disordered proteins, such as Tau or amyloid-β (Aβ) in the case of Alzheimer’s disease (AD)^1^ and α-Synuclein (αS) in Parkinson’s disease (PD). Tau, a microtubule-binding protein consisting of 441 residues, exhibits six isoforms in the human brain, with three isoforms containing three microtubule-binding repeats (3R) and the other three containing four repeats (4R).^1^ The presence of different isoforms in Tau filaments can lead to distinct Tau folds, contributing not only to AD but also to conditions like Pick’s disease^2^ or familial British dementia,^3^ among others. Similarly, different assembly modes of the 140-residue αS lead to diseases such as Multiple System Atrophy (MSA)^4^ or dementia with Lewy bodies (DLB).^5^ Aβ, another crucial protein implicated in AD,^6^ comprises 39–43 residues and typically adopts a random coil structure in solution.^7^ However, in AD patients, Aβ peptides form fibrils characterized by stacked β-sheets.^8^ The terms “tauopathies” and “synucleinopathies”^9^ have been introduced to categorize diseases resulting from abnormalities in Tau or αS, which can stem from various factors including posttranslational modifications^10^ or specific mutations,^11,12^ or through changes in environment.

The disease-related fibril formation for Tau, Aβ, and αS often takes place via the formation of structurally diverse oligomers.^13–15^ The transient nature of these oligomers makes their characterization challenging, which in turn complicates the identification of suitable molecules capable of preventing protein aggregation. Additionally, substantial structural polymorphism even in their aggregated states^16^ makes them largely inaccessible to conventional structural biology methods,^17,18^ leading them being frequently labeled “undruggable”.^19^ Efforts have been made to focus on specific segments of the fibril structure. For example, characterization of the fibril structure formed by the residues VQIVYK from the third repeat (R3)^20^ of Tau has enabled the development of inhibitors that prevent Tau aggregation.^21,22^ However, the inhibitors developed based on the particular residue segment have been found to be incapable of preventing the aggregation of full-length Tau.^22^ Although different studies have been carried out to develop ligands capable of preventing Tau aggregation,^23,24^ the concentration required for their effectiveness remains high, making them clinically less effective. Consequently, alternative strategies that shift the conformational landscape of these proteins away from aggregation-prone states represent a promising avenue for therapeutic intervention in neurodegenerative disorders.

In this context, spermine (Spm) represents an intriguing modulator. Among the different naturally occurring polyamines involved in crucial biological processes within cells,^25,26^ Spm possesses the highest charge of +4 and is known to be the most effective for the condensation of other biomolecules.^27^ Spm and spermidine have been found to contribute to neuroprotection by stimulating autophagic pathways that degrade damaged organelles and toxic protein aggregates.^28^ The role of spermine and other polyamines in the aggregation behavior of Tau, αS, and Aβ has been well-studied over the years.^29–31^ While the presence of Spm has been shown to decrease lag and transition times of the aggregation process for both αS^30^ and Aβ,^31^ higher-order polyamines have been shown to prevent fibrillization of Tau.^32^ These opposing effects have motivated therapeutic interest in modulating polyamine metabolism, particularly as a means of mitigating Tau aggregation.^33^

In a recent study, we demonstrated that Spm promotes liquid–liquid phase separation (LLPS) of Tau and αS, leading to the formation of highly dynamic condensates that facilitate autophagic clearance and suppress toxicity at the cellular level.^34^ While these results established the functional consequences of Spm-induced condensation, they did not resolve the molecular mechanisms by which Spm reshapes IDP conformational ensembles or explain why similar condensation behavior can lead to distinct aggregation outcomes for different IDPs.

Here, we address this unresolved question by developing an atomistic framework that directly links Spm-induced changes in monomeric conformational ensembles to disease-relevant fibril architectures. Using all-atom explicit-solvent molecular dynamics simulations, we assess whether Spm promotes or suppresses fibril-like contact formation in three aggregation-prone proteins: the K18 region of Tau, αS, and the amyloid-β (Aβ40), with patient-derived fibril structures serving as references. The validation of our results using ThT fluorescence experiments demonstrates a clear correlation between fibril-like contacts in the monomeric state and the overall aggregation propensity of these IDPs. Our results reveal that although Spm promotes condensation in both Tau and αS, its effects on aggregation-prone interactions are strongly protein sequence- and residue-specific. In particular, Spm disrupts key fibril-like contacts in K18, while enhancing aggregation-associated interactions within the aggregation-prone region of αS, thereby demonstrating that mesoscale phase behavior alone does not uniquely determine aggregation fate. For Aβ40, we identify the region-specific modulation of fibril-like contacts that drive aggregation in the presence of Spm. By bridging the effects of Spm on intramolecular interactions with disease-relevant fibril structures, this work provides a mechanistic link between small-molecule modulation, phase separation, and pathological aggregation.

## Results

### Protocol for Investigating Spermine-Mediated Modulation of Disease-Relevant Structures

In this study, we investigated the effect of Spm (structure shown in Figure S1) on the three disease-relevant IDPs: K18 region of Tau (hereafter K18), αS, and Aβ40 using all-atom MD simulations (see Methods). For each system, the details of protein:Spm ratios and their corresponding simulation details such as box size, number of atoms, and simulation lengths are given in Table S1 in SI. Additional simulation details are provided in the Methods section. To validate our simulations, we compared calculated ^15^N NMR chemical shifts for K18 (with and without Spm) with experimental data.^34^ The chemical shifts were computed using SPARTA+,^35^ based on snapshots extracted from the trajectories. The computed values of ^15^N chemical shifts in both the presence and absence of Spm are shown in Figure S2 in SI. The overall patterns suggest good qualitative agreement between simulation and experiment, though a few residues exhibited slightly higher deviations between the two conditions, likely arising from the intrinsic uncertainties in calculating chemical shifts from protein conformations.

Following the simulations, we investigated how Spm modulates the formation of disease-relevant structural motifs from these proteins. Recent studies have reported that intrachain hairpin formation correlates with the aggregation kinetics of Tau^36^ and Aβ,^100^ and structural analyses of early β-sheet oligomers in αS indicate that key fibrillar interactions can be encoded within hairpin-like conformations,^37^ linking monomer conformation to fibril assembly. Therefore, we employed a novel analysis protocol that quantifies Spm-induced promotion or suppression of fibril-like contact formation by using patient-derived fibril structures as structural references. The workflow of this protocol is schematically illustrated in Figure 1. At first, the proteins and Spm have been combined at different ratios, and the ensemble has been obtained using the MD simulations, as can be seen from Figure 1A. From these ensembles, the differences in the intraresidue contact map were obtained by comparing simulations with and without Spm, allowing us to identify the contacts that increase or decrease in the presence of Spm (Figure 1C). Simultaneously, different fibril structures associated with these proteins have been collected to obtain the native contacts within the disease-relevant structures (Figure 1B). The presence of diverse filament morphologies in Tau,^38^ αS, and similar proteins indicates that multiple aggregate conformations can exist. Moreover, both Tau and αS filaments from patients have been found to promote disease-relevant fibril formation more effectively than in vitro–assembled filaments,^39^ underscoring the biological relevance of disease-specific polymorphs. To capture this diversity, we selected fibril structures directly derived from patient brain tissues for Tau, αS, and Aβ40. Given the structural heterogeneity observed even among filaments associated with the same disease, we first clustered the collected fibril structures based on shared intrachain contacts. A few differently structured Tau filaments are shown in Figure 1B, which have been used for clustering and finding the native contacts along with several other reported structures. One such contact map corresponding to a Tau fibril structure is shown in Figure 1D.

**Figure 1:**
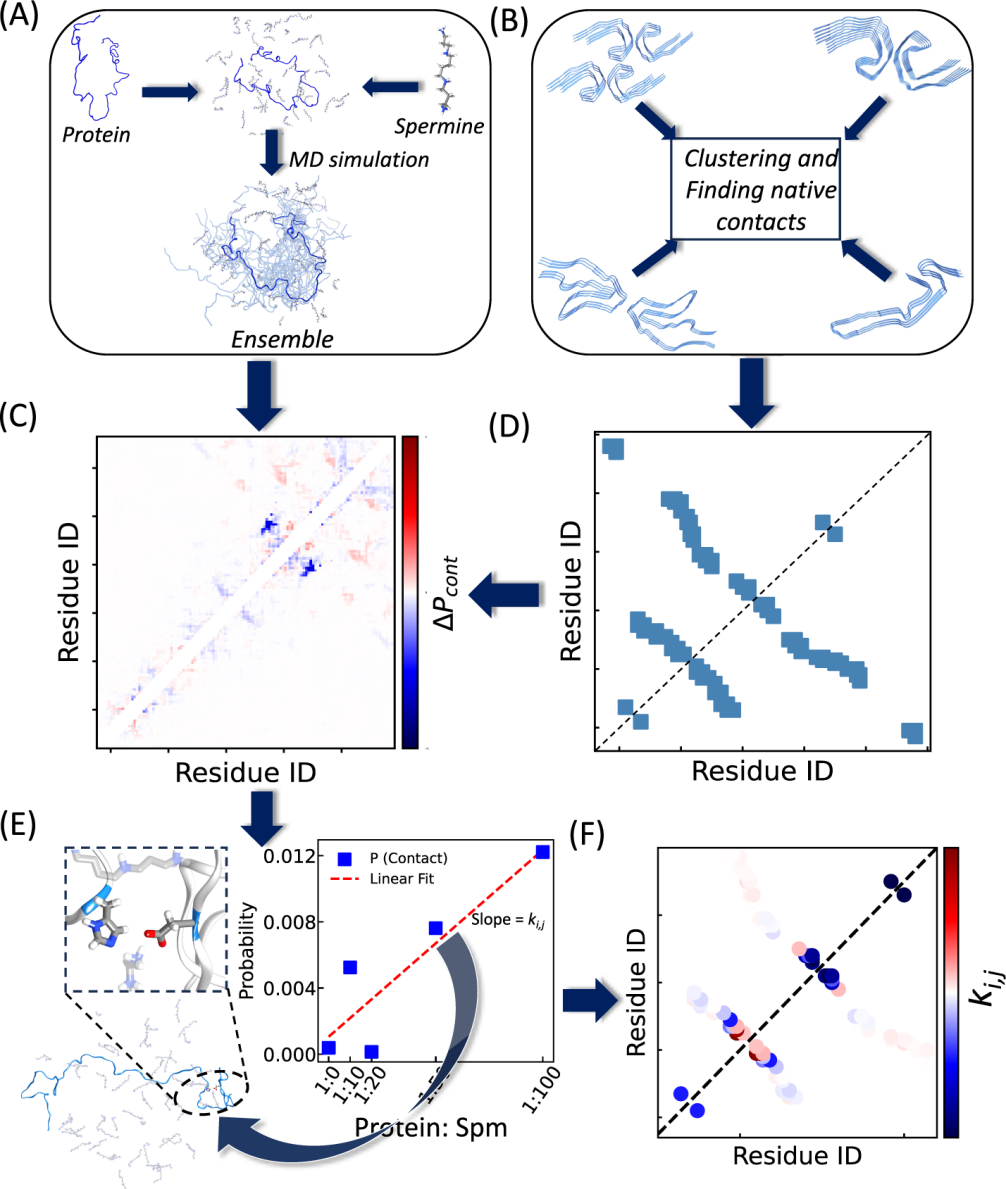
Schematic diagram of the protocol to investigate the influence of spermine (Spm) on disease-relevant fibril structures. In panel (A), IDPs are combined with Spm, and molecular dynamics simulations are performed to generate the structural ensemble. From these ensembles, changes in protein contact maps between the absence and presence of Spm are calculated (panel C). Panel (B) shows representative Tau fibril structures, which are clustered to identify native contacts. One example of a contact map from a fibril structure is shown in panel (D), highlighting residue pairs involved in fibril formation. In panel (E), one such contact between residues 330 and 359 observed in the simulation of K18 is illustrated, along with its probability *P_i,j_* at different Spm ratios. For each fibril-native residue pair, the modulation coefficient *k_i,j_* is obtained as the slope from linear regression of the contact probability *P_i,j_* versus Spm ratio. Using this approach, *k_i,j_* of every fibril contact pair is quantified, producing the contact modulation map shown in panel (F).

For K18 of Tau, among the different structures observed at different conditions, we focus on filaments associated with AD,^40^ corticobasal degeneration,^41^ and Pick’s disease.^2^ The Tau inclusions observed in the Cryo-EM structure reported from AD patients^40^ exhibit polymorphism in the paired helical filaments (PHFs) and straight filaments (SFs)^42^ whose cores are made up of double helical stacks of C-shaped subunits as seen previously for other Tau filaments.^43^ With only a few residues present in these units, large segments forming the N-terminal domain (NTD) and C-terminal domain (CTD) of Tau remain disordered in these structures and form a fuzzy coat.^44^ However, while the structure reported for Pick’s disease^2^ does not have the second repeat (R2) and does not form C-shaped subunits, the filaments related to CBD^41^ are not found as double helical stacks. The fact that the filaments associated with AD differ in symmetry between PHFs and SFs^40^ further adds to the complexity involved in the structures of Tau inclusions. Therefore, in addition to the above-mentioned structures, other similar structures reported elsewhere^45,46^ are also included for Tau to find the pairs that are present in fibril structures. For αS, the filament structures chosen here are the ones reported from patients having PD^47^ and MSA.^48^ The αS filaments found in these structures and others obtained in vitro^49^ extend from residues 30 to 100. The PDB IDs belonging to Tau and αS used here are listed in Tables S2 and S3, respectively. For Aβ40 too, structural polymorphism becomes evident from the difference in the β-sheet twist found in the filaments obtained in vitro^50^ and in the one found in AD patients.^51^ Therefore, we select two structures found from AD patients^51,52^ to check whether Spm promotes the contacts found in this conformation or not. For Tau, clustering of 44 filament structures yielded 14 distinct clusters with two clusters containing 13 members each and seven unique structures showing no shared contacts. The list of these fibril structures and their corresponding cluster IDs is given in Table S2. The contact maps for these clusters are shown in Figure S3. For αS, 5 clusters were identified from 7 filament structures (Figure S4). The list of the 13 αS fibril structures is given in Table S3 along with their corresponding cluster IDs.

Using disease-relevant fibril structures determined experimentally and contact maps obtained from all-atom simulations at different Spm concentrations, we would like to assess whether the intrachain contacts observed in the fibril structure are preserved or altered in simulations with varying Spm concentrations. For example, the contact between residues 330 and 359 belonging to the Tau-K18 system is shown in a simulation snapshot in Figure 1E which is observed in numerous fibril structures. For this contact pair, its probabilities at various Spm concentrations have been checked to see whether this contact formation is more or less probable in the presence of Spm. Despite the intrinsic variability of IDP simulations, the modulation coefficient (*k*_*i,j*_), representing the slope from linear regression of contact probabilities versus Spm ratios, captures whether Spm systematically enhances or suppresses specific contacts. Mapping *k*_*i,j*_ for all fibril-native residue pairs yields the contact-modulation map shown in the bottom-right panel of Figure 1F. This same protocol has been applied to different disease-causing fibril structures belonging to the three IDPs studied here.

### Divergent Mechanisms of Spermine Modulation in K18, αS, and Aβ

In Figure 2A-C, we check Spm’s influence on the propensity to form filaments related to the three IDPs. The sequences for these proteins are shown in the left panels. The K18 segment (residues 244–372) shown in Figure 2A is the microtubule-binding region of the longest human Tau isoform (441 residues). Four repeat regions within K18 denoted as R1, R2, R3, and R4 (shown in different colors in Figure 2A) form the core of the cross-structure of Tau-paired helical filaments.^53^ The number of positively and negatively charged residues shown with blue and red lines, respectively, on the sequence indicates a net positive charge for this system which is likely to create an overall repulsive electrostatic interaction between K18 and Spm. We check how Spm modulates the overall changes in probabilities for the contacts observed in the filament structure belonging to AD, using the cryo-EM structure (PDB ID: 5O3L).^40^ The fibrillar assembly is shown in the middle panel of Figure 2A, with one monomer highlighted in a darker shade and zoomed in to illustrate intrachain interactions. The contact modulation map on the right with the red and blue dots obtained using the protocol shown in Figure 1 gives an indication of whether the contact probabilities increase or decrease, respectively, upon Spm interaction. Similar analyses for CBD and Pick’s disease are presented in Figures S5 and S6, along with the respective filament structures and magnified monomer views.

**Figure 2:**
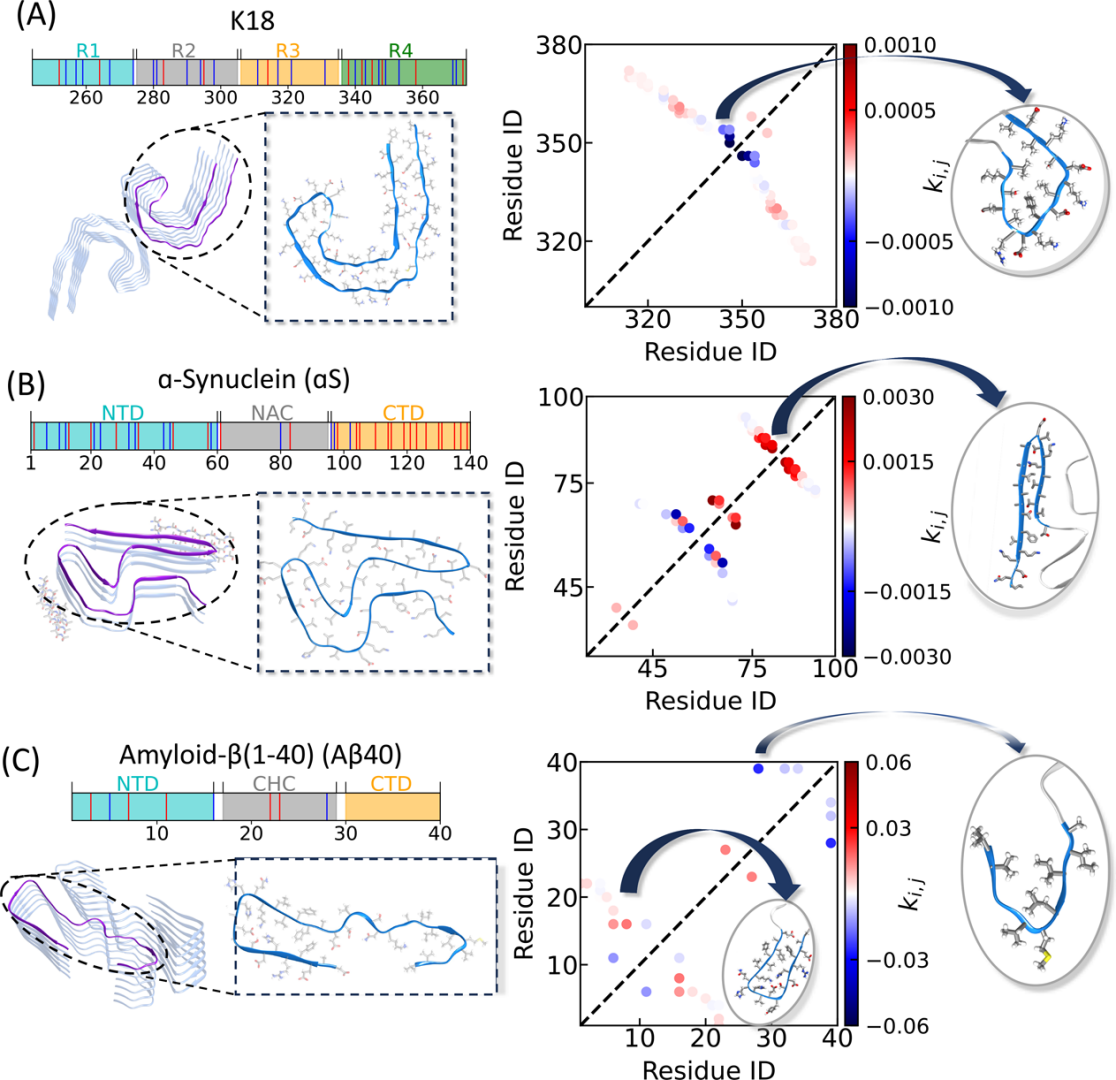
Influence of Spm on the propensities to form disease-relevant native contacts in Tau (A), αS (B), and Aβ40 (C). The top panels on each of the three subfigures display the sequences of the three IDPs. The bottom-left panels show the corresponding fibril structures, including Tau (PDB ID: 5O3L), αS (PDB ID: 8A9L), and Aβ40 (PDB ID: 6SHS), with one representative monomer highlighted in each case. The right panels present the contact modulation maps for the three systems calculated using the protocol shown in Figure 1. The insets highlight regions that exhibit pronounced alterations in contact formation upon Spm addition.

From the pattern in Figure 2A, we observe that Spm leads to a decrease in fibril-like contacts between residues 340 and 345, indicating specific binding of Spm at this region. This region corresponds to one end of the C-shaped helical subunit, where charged and hydrophobic residues are densely packed, as illustrated in the inset on the bottom-right of Figure 2A. This central turn, comprising a heterosteric hydrophobic zipper, has been proposed to form the monomeric hairpin that seeds filament formation in sporadic AD.^36^ Further, this segment has been found to be crucial for governing temperature-dependent conformations of Tau.^54^ Since Spm binds near this region and disrupts key intrachain contacts, it may inhibit the aggregation of Tau into disease-relevant fibrils. For the CBD-related structure (Figure S5), we also observe a predominance of blue dots, suggesting that Spm interferes with contacts essential for filament formation in this case as well. In contrast, the pattern for Pick’s disease (Figure S6) is less pronounced, possibly due to the absence of the R2 repeat in this isoform, whereas the Tau sequence used in our simulations includes all four microtubule-binding repeats. Together, these results highlight that Spm engages in specific interactions within the turn region of the Tau fibril structure, thereby perturbing a structural element critical for filament nucleation. To evaluate the robustness of this observation with respect to fibril polymorphism, we repeated the contact modulation analysis for additional Tau fibril structures representing distinct structural clusters (Figure S7). In all cases, Spm reduces fibril-native contact probabilities within aggregation-relevant regions, although the magnitude of the effect varies among polymorphs. These results indicate that the qualitative trend of Spm-mediated disruption of fibril-like contacts is not specific to a single fibril model but is preserved across structurally diverse Tau polymorphs. Importantly, while our previous work showed that Spm promotes the formation of dynamic, liquid-like Tau condensates,^34^ the present atomistic analysis suggests that Spm simultaneously suppresses fibril-like interactions within these condensates at early stages, thereby decoupling phase separation from pathogenic aggregation.

The sequence of αS (Figure 2B) consists of a positively charged NTD, a nonamyloid-β component (NAC) region, and a highly negative CTD as can be seen from the different color code. The presence of several negatively charged residues (red lines) in the sequence is likely to make its interaction with Spm attractive. For this system, we analyzed the associated filament structure (PDB ID: 8A9L^47^), shown in the bottom-left panel of Figure 2B. The contact modulation coefficients *k*_*i,j*_, obtained using the protocol described in Figure 1, are mapped onto the corresponding monomer structure and represented in the contact map on the right. In contrast to the Tau filaments, αS exhibits a pronounced increase in contact probabilities, particularly within the NAC region, as highlighted in the inset on the right. A similar trend is observed for the MSA-related filament structure (PDB ID: 6XYO^48^), presented in Figure S8. Interestingly, despite the structural differences between PD- and MSA-associated αS filaments, the Spm-induced increase in contact probability localizes to the same NAC region in both structures. A previous study on αS and Spm using paramagnetic relaxation enhancement and NMR dipolar couplings indicated that Spm binds primarily to the CTD and weakens its long-range interaction with the NTD and NAC region.^55^ This release likely renders the NAC more accessible for intermolecular contacts. Consistent with this, our data suggest that Spm enhances intrachain contacts within the NAC itself, implying a more global reorganization in contrast to the local structural perturbation observed in Tau.^36^ In principle, this shift promotes in general condensation, including both aggregation and phase separation. Indeed, in our previous study, we demonstrated that Spm extends the lifespan of a *C. elegans* PD model in a concentration-dependent manner by promoting αS LLPS, which facilitates autophagosome-mediated clearance.^37^ This delicate balance between aggregation and LLPS—also observed under varying salt concentrations^56^—highlights a regulatory role of Spm in tuning the assembly state of αS.

The Aβ40 sequence shown in Figure 2C has an overall −3 net charge and, therefore, is expected to have a net attraction with Spm. The sequence features a CTD, NTD, and the central hydrophobic core (CHC), each highlighted with different colors. For Aβ40, we examined the Alzheimer’s disease–associated fibril structure (PDB ID: 6SHS^51^), shown on the bottom-left panel of Figure 2C with the intrachain contacts within one monomer zoomed in. The corresponding *k*_*i,j*_ values, obtained using the protocol shown in Figure 1, are shown in the right panel of Figure 2C. The contact difference map reveals a heterogeneous response: within the CHC and parts of the NTD, Spm enhances several contacts (see insets), while in the CTD and other aggregation-prone regions, Spm reduces contact probabilities. Thus, rather than uniformly stabilizing or destabilizing fibril-like interactions, Spm reshapes the balance of intrachain contacts across Aβ40 in a region-dependent manner. The experimentally observed increase in Aβ40 aggregation in the presence of Spm^31^ indicates that the promotion of specific contacts outweighs the loss of others, thereby dominating the overall behavior of Aβ40 in solution. A similar behavior is observed for another Aβ40 filament structure (PDB ID: 8QN6 ^52^) which has similar structural features as the one shown in Figure 2C indicating consistent yet regionally distinct effects of Spm across Aβ40 filaments.

These results highlight a divergent influence of Spm on different amyloid systems: it promotes filament-like intrachain contacts in αS (consistent with previous observations^30^), disrupts key nucleation-region contacts in K18, and exerts mixed, region-specific effects in Aβ40. Experimental studies of αS^30^ and Aβ^31^ have already demonstrated Spm’s ability to modulate their aggregation, whereas for K18 this remains less explored. Our simulations provide a clear, testable hypothesis that Spm should inhibit K18 aggregation, making this construct an ideal system to validate our computational framework. We therefore next assessed the impact of Spm on K18 aggregation using Thioflavin T (ThT) fluorescence assays.

### Experimental Validation of Spermine-Mediated Inhibition of Tau Aggregation

To validate our simulation-based predictions, we monitored the aggregation kinetics of the K18 repeat domain as well as full-length Tau using ThT fluorescence assays in the absence and presence of increasing Spm concentrations (Figure 3). Aggregation reactions were carried out in 25 mM HEPES buffer (pH 7.4) at 37 °C with agitation, and the ThT signal was recorded over time to capture the fibril formation. Although the all-atom MD simulations were performed at temperatures consistent with NMR measurements, the ThT assays at physiological temperatures allow us to assess whether the qualitative direction of Spm-induced modulation of aggregation is preserved under different conditions. The kinetic traces were fitted to a sigmoidal function given in the Methods section, allowing us to extract phenomenological parameters such as the maximum fluorescence intensity (*F*_max_) and half-time (*t*_1/2_) of aggregation. This approach provides a quantitative comparison of how Spm influences fibril growth across different Tau constructs.

**Figure 3:**
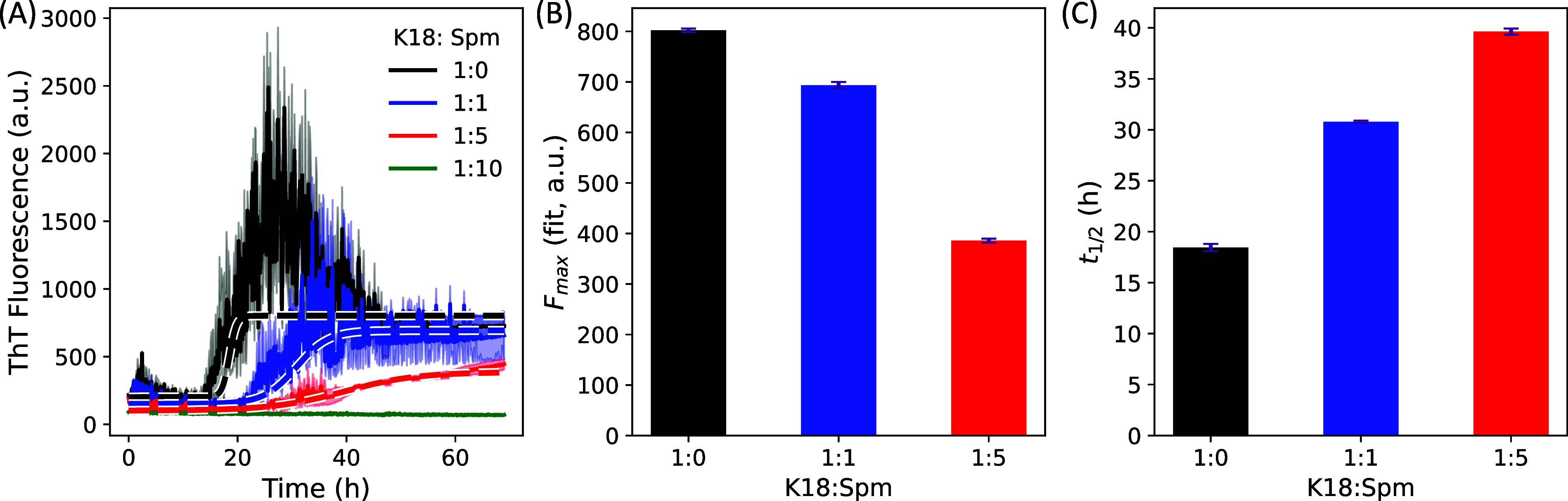
Amyloid aggregation kinetics of K18 monitored by ThT fluorescence in the presence of spermine. ThT fluorescence measurements were performed using 20 μM K18 at varying Spm ratios, as indicated in the legend. (A) Time-dependent ThT fluorescence of K18 in the absence and presence of Spm. Solid lines represent the mean of three independent measurements, with the standard error of the mean shown as light-colored shading. Dashed lines indicate sigmoidal fits. For the highest Spm concentration, fitting was not performed. (B, C) Fitted values of the maximum fluorescence intensity (*F*_max_) and aggregation half-time (*t*_1/2_), respectively, at different Spm concentrations. The uncertainty in the fitted parameters was estimated by using a bootstrapping procedure.

Figure 3A shows clear concentration-dependent inhibition of K18 aggregation by Spm. ThT fluorescence intensity progressively decreases with increasing Spm concentration, reaching minimal signal (no aggregates) at the 1:10 K18:Spm ratio, indicating strong suppression of fibril formation. Sigmoidal fits (dashed lines; see Methods) were applied to the 1:0, 1:1, and 1:5 K18:Spm systems, while the 1:10 condition without discernible aggregation was not fitted. The fitted parameters show a systematic reduction in the maximum fluorescence intensity (*F*_max_; Figure 3B) accompanied by a substantial increase in aggregation half-time (*t*_1/2_; Figure 3C), which more than doubles at the 1:5 K18:Spm ratio. These results indicate that Spm markedly slows aggregation kinetics and lowers the fibril yield. Consistent with our simulations, this inhibition likely arises from a modest reduction in fibril-prone intramolecular contacts within the K18 monomer, leading to an overall decrease in the aggregation propensity.

It is known that truncation of the N- and C-terminal regions of Tau enhances its aggregation propensity.^57^ To assess whether Spm exerts a comparable effect on the aggregation behavior of full-length Tau, parallel experiments using the same protein-to-Spm ratios as for K18 have been carried out on the 441-residue Tau. The fluorescence curves shown in Figure S9 indicate a much slower aggregation process for the full-length Tau in the absence of Spm compared to K18. Such slower aggregation is expected as the N- and C-terminal regions act as a fuzzy coat next to the aggregation core of Tau. However, in the presence of Spm, we see a similar decrease in the fluorescence intensity as observed for K18. This indicates that the subtle changes in the contact formation shown in Figure 2A can also impact the aggregation propensity of full-length Tau. These results demonstrate that Spm interferes with the amyloid formation process and further underscore the greater susceptibility of the nucleation-competent K18 construct to Spm-mediated inhibition.

These experiments provide direct validation of our computational framework, establishing K18 as a tractable system in which Spm disrupts aggregation kinetics, in agreement with our predictions. Having confirmed this inhibitory effect experimentally, we next turned back to simulations to examine in greater detail how Spm regulates the conformational ensemble of disordered proteins and thereby modulates their aggregation propensities.

### Global Structural Modulation of K18, αS, and Aβ40 by Spm

Having established experimentally that Spm inhibits Tau aggregation, we next investigated how Spm regulates the conformational ensembles of different amyloidogenic proteins at a global scale. To this end, we first characterized structural features that capture overall compaction and chain organization, including radial distribution functions, the radius of gyration, and secondary structure preference. These analyses provide a comprehensive view of how Spm reshapes the global conformational behavior of K18, αS, and Aβ, before moving on to residue-specific interactions. Due to the high net charge on Spm, the electrostatic interactions between Spm and proteins are likely to play a pivotal role in governing their behavior. To probe this, we analyzed the radial distribution function (RDF), *g*(*r*), which quantifies the spatial distribution between protein C_α_ atoms and the heavy atoms of Spm for different protein:Spm ratios. The *g*(*r*) plots for the three systems, presented in Figure 4A, reveal distinct interaction patterns. As expected for K18 with a net positive charge of +10, the RDFs indicate weak and unfavorable interactions with Spm, evident from the low peak heights in the left panel of Figure 4A. Conversely, αS with a negative charge of −9 shows strong and favorable interactions with Spm, as seen in the middle panel of Figure 4A. Notably, the peak heights decrease with increasing Spm concentration, suggesting a saturation of interactions between αS and Spm. Similarly, Aβ40, which also has a net negative charge of −3, exhibits favorable interactions with Spm as can be seen in the right panel of Figure 4A.

**Figure 4:**
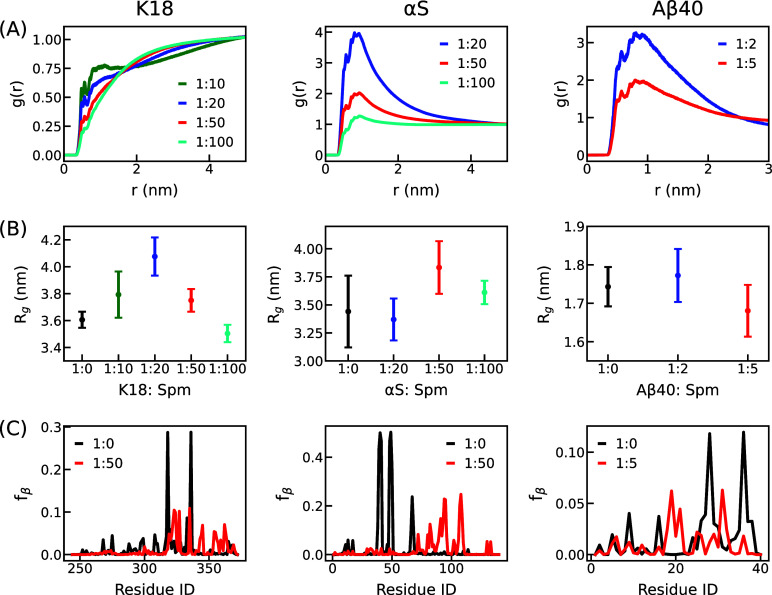
Properties of K18, αS, and Aβ40 in the absence and presence of Spm. (A) The radial distribution function, *g*(*r*) vs distances at different Spm concentrations. The *g*(*r*) value has been calculated with respect to the C_α_ atom of all residues from the three systems and the heavy atoms of Spm. (B) The *R*_g_ values with respect to Spm ratios for all the systems. (C) The fraction of β-sheet conformation, *f*_β_, for each residue without Spm (black line) and with Spm (red line), for the three systems.

Based on these interaction patterns, one might expect the radius of gyration (*R*_g_) of the protein systems to mirror the trends observed in *g*(*r*): attractive association with Spm should neutralize electrostatic repulsion and promote chain compaction. However, the calculated *R*_g_ values reveal more complex behavior and in some cases they do not consistently align with the RDF trends, as shown in Figure 4B. First, for K18, the *R*_g_ value in the absence of Spm closely matches the experimentally reported value of 3.8 ± 0.3 nm,^58^ even though the experimental temperature is slightly higher than the one used here. When Spm is introduced, *R*_g_ for K18 initially increases with Spm concentration, as binding to negatively charged residues enhances the net positive charge of K18 and strengthens intrachain electrostatic repulsion. However, at higher Spm concentrations, *R*_g_ returns to values comparable to those of the no-Spm condition, since excess Spm screens the electrostatics and limits further charge imbalance. Notably, as K18 expansion has been associated with increased aggregation propensity,^59^ these *R*_g_ trends imply that Spm-mediated inhibition of Tau aggregation is not a direct consequence of structural compaction induced by Spm binding, but instead point to our earlier observation that localized specific Spm–protein interactions may be at play.

In contrast to K18, for αS, the *R*_g_ value without Spm is close to the previous FRET measurement of 3.3 ± 0.3 nm^60^ and smaller than the previous SAXS measurement of 4.0 ± 0.1 nm,^61^ which could be attributed to the temperature and ionic strength differences between the simulation and different experimental setups. With Spm, *R*_g_ remains largely unchanged at lower Spm concentrations but increases at higher ratios, reflecting a moderate structural expansion. This trend can be explained by the neutralization of negative charges in the CTT, which reduces its long-range attraction to the NTD. Unlike K18, αS does not exhibit a turning behavior, likely because its strong net negative charge prevents saturation within the concentrations tested, so Spm continues to neutralize charges rather than acting as a general electrolyte that primarily provides screening. Meanwhile, Aβ40 exhibits minimal variation in *R*_g_ across all Spm concentrations, as shown in the right panel of Figure 4B. Although Aβ40 demonstrates favorable interactions with Spm, these interactions do not translate into chain expansion. Further insights into these behaviors come from the distributions of *R*_g_ values, as shown in Figure S10. The plot reveals that the *R*_g_ distributions for all three systems are broadly similar for K18 and Aβ40, irrespective of the Spm presence. However, the distribution shifts slightly to the right at higher Spm ratios for αS. Collectively, these results underscore the complexity of interactions between Spm and these protein systems and highlight the role of specific interactions beyond mere electrostatic effects.

Despite the observed differences in *R*_g_ patterns among the three systems, a unifying trend emerges for another structural property, the Flory scaling exponent, ν.^62,63^ As shown in Figure S11, we fitted the root-mean-squared intrachain distances as a function of the sequence separation |*i*–*j*| to obtain ν for each case using a constant prefactor of 0.55 nm as determined from previous literature.^64,65^ Table S1 lists ν values for K18, αS, and Aβ40, with and without Spm. In the absence of Spm, ν values of 0.59, 0.57, and 0.58 for K18, αS, and Aβ40, respectively, indicate that all three systems adopt extended conformations in solution. Upon introduction of Spm, ν either increases or remains largely unchanged across all systems, irrespective of their net charges, suggesting an overall elongation of the proteins. This observation implies that Spm, despite being positively charged, can interact with the positively charged K18 in a manner that promotes structural elongation.

We next performed secondary structure analysis to check whether a residue on average remains in a helical (H), β-sheet (β), or random coil (C) conformation. Notably, the β-sheet conformation has been implicated in the aggregation behavior of Tau,^53^ αS,^66^ and Aβ40.^67^ Thus, Spm-induced modulation of secondary structure propensities may explain its effects on aggregation. To assess this, we calculated the secondary structure propensities of individual residues in simulations with and without Spm. The DSSP module^68^ of GROMACS has been applied on the monomeric MD trajectories to assess the per-residue secondary structure propensities. For all three systems, the fraction of β-sheet conformation (*f*_β_) is shown in Figure 4C. For clarity, we only show the probabilities for the no-Spm systems and the systems with a protein:Spm ratio of 1:50 for K18 and αS, and 1:5 for Aβ40. The corresponding fractions of coil (*f*_C_) and helical (*f*_H_) conformations are provided in Figure S12. For K18 (left panel, Figure 4C), regions with higher extended-state propensity qualitatively agree with previous NMR results.^36^ Among these, the 335–340 segment, which is critical for disease-relevant filament formation as observed in Figure 2A, shows a notable decrease in *f*_β_ upon Spm addition, suggesting a direct influence of Spm on local conformational preferences. For αS (middle panel, Figure 4C), we observe that in the absence of Spm, residues 36–42 exhibit high *f*_β_, consistent with their known role in aggregation and function.^69^ Upon Spm binding, *f*_β_ decreases in this region but increases within the NAC region, where Spm also enhances disease-relevant contacts, pointing to a functional correlation. In Aβ40 (right panel, Figure 4C), *f*_β_ decreases in the NTD and increases in the CHC upon Spm addition—mirroring the contact changes observed earlier. These findings underscore the role of extended conformations in promoting aggregation-prone interactions, even at the monomer level.

The three systems highlight distinct behaviors in the presence of Spm: K18 undergoes nonmonotonic change in *R*_g_, reflecting initial binding and subsequent saturation and screening; αS displays progressive expansion due to charge neutralization without saturation; and Aβ40 shows limited compaction. These observations suggest that specific amino acid interactions and conformational rearrangements in particular regions rather than overall net charge and global chain dimensions play a decisive role in determining structural outcomes. To dissect these effects in greater detail, we next examined residue-specific interaction patterns with Spm and their consequences on the overall contact maps.

### Residue-Specific Interaction Patterns Underlying Spm-Mediated Modulation

In this section, we explore the specific interactions between Spm and the three systems as well as the structural and conformational outcomes of these interactions. We start by computing the average number of Spm molecules interacting with each protein residue. This metric, denoted as *N*_Spm_, has been plotted against residue indices for K18, αS, and Aβ40 in the left, middle, and right panels of Figure 5A, respectively. The different regions in the sequences are also shown at the top for the sake of convenience. Across all three systems, prominent *N*_Spm_ peaks are observed near negatively charged residues, reflecting the electrostatic nature of Spm–protein interactions. For K18, multiple peaks appear near the start of the R4 repeat, a region enriched with negatively charged residues. This interaction likely explains the observed increase in *R*_g_ at lower Spm concentrations. The ^332^PGGG^335^ motif near this site has been found to be crucial for forming the PHFs of Tau.^40^ Binding of Spm near these residues is likely to expand the structure of K18 and is likely to be crucial for preventing the formation of a hairpin bend required for fibril assembly. In αS, Spm predominantly interacts with the negatively charged N-terminal region, consistent with its high charge density. While weaker interactions are seen at other negatively charged sites, these are less frequent—aligning with prior NMR chemical shift data for this system.^47^ For Aβ40, *N*_Spm_ peaks appear at both termini and the central region, again correlating with the positions of negatively charged residues. The binding patterns observed here suggest that the changes in the extended-state conformation seen in Figure 4C are a direct consequence of Spm interactions. However, the primary binding sites identified in our simulations differ from those showing the largest chemical shift perturbations in earlier NMR studies.^31^ To reconcile this, we next examine changes in intrachain contact maps upon Spm addition, aiming to clarify how Spm binding translates into conformational alterations across these systems.

**Figure 5:**
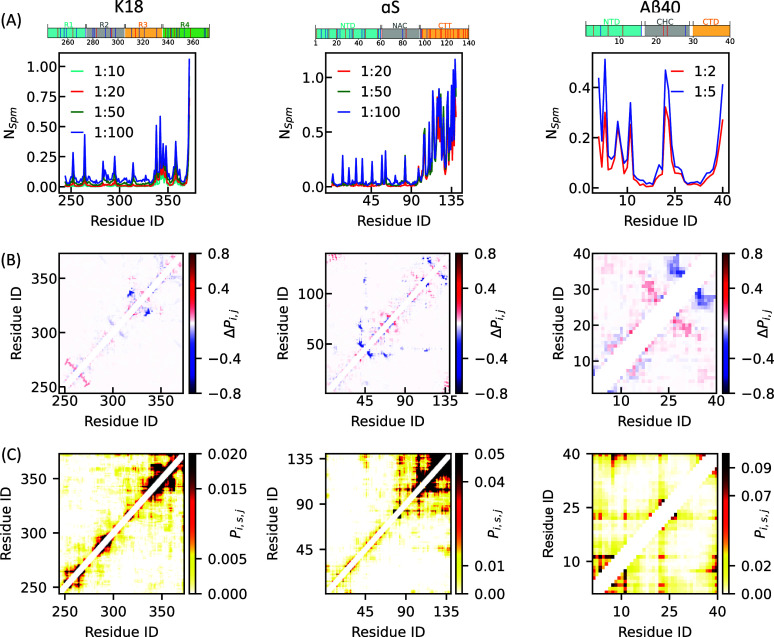
Spermine interactions with K18, αS, and Aβ40 and their effects on intraresidue contacts. (A) Average number of Spm molecules (*N*_Spm_) near each protein residue. (B) Changes in intraprotein contact probabilities (Δ*P*_i,j_) for residue pairs, calculated between Spm-free and Spm-bound simulations (1:50 protein-Spm for K18 and αS; 1:5 for Aβ40). (C) Spm-mediated contact probabilities (*P_i,s,j_*) for residue pairs separated by more than three amino acids, measured under the same protein-Spm ratios as in (B).

To investigate how Spm affects intrachain contacts within these systems, we analyzed contacts between heavy atoms of residue pairs separated by at least three residues, both in the presence and absence of Spm. We then computed the difference in contact probabilities by subtracting the values in the Spm-free system from those in the Spm-containing system, indicating whether Spm promotes or disrupts intramolecular contacts. For K18 and αS, the changes in contact probabilities between the Spm-free system and the 1:50 protein–Spm condition are shown in the left and middle panels of Figure 5B, respectively. For Aβ40, the comparison is between the 1:5 protein–Spm and the Spm-free system, shown in the right panel of Figure 5B. In K18, the most pronounced reduction in contacts occurs near residues 335–340, coinciding with the region of highest Spm-binding propensity. A smaller decrease is observed near residue 310. Similar behavior has been observed for other Spm ratios also, as shown in Figure S13. These changes are likely linked to Spm’s ability to modulate Tau aggregation. Prior studies have reported differential interactions between the R1–R4 repeat domains of Tau,^20,70–73^ with the R3–R4 segment forming the core of protofibrils in patients with chronic traumatic encephalopathy (CTE).^71^ Spm interaction near the start of the R4 region could therefore influence these aggregation patterns. Notably, the amyloidogenic motif ^306^VQIVYK^311^, located within the R3 repeat, has been shown to drive amyloid formation in vitro^20^ and contribute to pathology in vivo.^70^ A reduction in intrachain contacts near this region suggests that the motif may lose its ability to engage upstream sequences, a process known to regulate Tau’s aggregation propensity.^72^ These findings indicate that despite the overall electrostatic repulsion between the positively charged K18 and Spm, Spm exhibits selective binding to critical regions in K18, modulating its intramolecular contacts and potentially influencing its aggregation into disease-relevant fibrils.

For αS, Spm shows changes in contacts at several places, with the most notable decrease in contacts at NTD and between NTD and CTD. The reduction in these long-range interactions likely drives chain expansion, consistent with the modest increase in the *R*_g_ observed in Figure 4B. Additionally, a slight increase in contacts has been observed at the NAC region across all the Spm ratios as can be seen in the middle panel of Figure 5B and in Figure S14. These changes resemble the effects seen upon truncation of the CTD,^74,75^ suggesting that Spm coats this region and thereby exposes the NAC domain, making it more susceptible to intra- and interchain interactions. Another notable contact change occurs near residue 46, a site implicated in fibril-like contact formation. The E46K mutation, which introduces a positive charge at this position, is known to enhance αS fibrillization.^76^ Since Spm is likely to bind at or near residue 46 and similarly alter local electrostatics, it may promote aggregation through a mechanism akin to that of the E46K mutation. Taken together, these contact-map changes provide a molecular explanation for the enhanced LLPS propensity of αS in the presence of Spm observed in our previous study.^34^ Specifically, Spm-induced disruption of long-range intramolecular interactions expands the αS chain while concomitantly exposing the aggregation-prone NAC region, thereby facilitating both phase separation and aggregation-prone interactions.

For Aβ40, the contact maps indicate a mixed pattern of changes upon Spm binding, as seen in the right panel of Figure 5B and in Figure S15. Notably, the CHC and CTD regions show distinct responses: intramolecular interactions within the CHC increase, whereas those within the CTD decrease. In addition, enhanced interactions are observed within the CHC, particularly between residues 16–20 and 27–30. This effect likely arises from Spm binding to residues ^22^Glu-^23^Asp (Figure 5A), which lie between the two fragments. By neutralizing negative charges, Spm reduces electrostatic repulsion within this region. The two fragments showing significant contact variations upon Spm addition align with a previous NMR study on Aβ40 with Spm, where residues with the largest chemical shift changes correspond to positions near residues 4–5, 15–17, and 27–28.^31^ Similar effects have also been documented in the presence of positively charged metal ions^77^ and other small molecules.^78^

In addition to analyzing changes in contact maps in the presence of Spm, we also examined Spm-mediated intrachain contacts in these systems. Figure 5C presents the Spm-mediated contact maps for K18 (left), αS (middle), and Aβ40 (right). For K18, the plot reveals that Spm-mediated contacts are predominantly short-range. Although a major change in contact probability is observed near residues 335–340, consistent with the region of strongest Spm binding, several upstream residues also display Spm-mediated interactions. In the case of αS, the most prominent Spm-mediated contacts occur within the CTD, consistent with the higher Spm binding observed in this region. Interestingly, we also detect long-range contacts between the NTD and CTD, likely facilitated by Spm. This interaction may disrupt normal interactions between the NAC region and NTD, thereby increasing the aggregation propensity of αS by freeing the NAC domain for self-association. For Aβ40, the most probable Spm-mediated interactions are found within the NTD and between the NTD and CTD. Although residues 23–24 interact with Spm, the right-side plot in Figure 5C clearly indicates that the interaction between the regions of NTD and CHC observed in Figure 5B does not come from Spm mediation but direct residue–residue interactions.

To determine whether the identified Spm interaction sites correspond to persistent binding or rapid exchange, we quantified two complementary residence times together with exchange rates (Figure S16, see Methods). The local residence time τ_residue_ measures how long an Spm molecule remains associated with a specific residue before it leaves that site. The protein-level residence time τ_protein_ measures, for an Spm molecule initially bound near a given residue, how long it remains associated with any part of the protein surface before fully dissociating. Across all systems, τ_residue_ values are short even at residues with high interaction probabilities, whereas τ_protein_ is modestly longer for αS and Aβ40. Importantly, residues with elevated Spm interaction probabilities exhibit higher exchange rates rather than prolonged local residence times, indicating that enhanced interactions arise from frequent transient binding events rather than stable complexes.

Together, these results demonstrate that Spm can facilitate both short- and long-range interactions within IDPs. In K18, the distribution of negatively charged residues drives Spm to bind regions critical for aggregation, potentially disrupting the key contacts. In αS, Spm binding at multiple acidic sites reduces the number of CTT-NAC interactions and increases the solvent accessibility of the aggregation-prone NAC. For Aβ40, aggregation of the CHC is normally hindered by surrounding acidic residues; however, Spm binding likely neutralizes electrostatic repulsion, enabling CHC-driven aggregation. These findings suggest that Spm modulates the aggregation of these three IDPs via distinct mechanistic pathways, each shaped by the protein’s charge distribution and structural organization, underscoring the need to consider sequence- and region-specific effects when studying polyamine–protein interactions.

## Discussion

Despite significant advances in experimental and computational studies, unraveling the drivers of IDP aggregation and identifying strategies to prevent it remain a formidable challenge. One promising avenue involves examining the influence of cellular components, which may indirectly modulate the formation of disease-relevant assemblies. Among these, polyamines represent a class of small, positively charged molecules that have been implicated in tauopathies and synucleinopathies. In this study, we investigate the impact of one such polyamine, spermine, on the aggregation behaviors of three representative IDPs: Tau, αS, and Aβ40. Using long-time scale all-atom explicit-solvent molecular dynamics simulations and ThT fluorescence assays, we probe how Spm affects the early stages of disease-relevant filament formation. Given the growing evidence that small oligomers may be toxic,^79^ and that aggregation may be initiated at the monomeric level,^36^ monomeric all-atom simulations provide a valuable lens to identify residue-specific interactions that shape the aggregation process.

To probe how Spm influences fibril-like interactions, we examined intrachain native contact propensities derived from fibril structures. Our findings show a reduction in contact probabilities for Tau filament structures associated with Alzheimer’s and Pick’s disease in the presence of Spm. Conversely, αS filament contacts linked to Parkinson’s and Multiple System Atrophy exhibited increased probabilities. Aβ40 displayed a mixed response. These observations were supported by ThT fluorescence experiments for K18, which showed a decreased level of aggregation in the presence of Spm. For αS, the enhancement of aggregation by Spm is consistent with prior findings. Although fibril formation is inherently a multimeric process, the present simulations isolate how Spm reshapes fibril-like conformational populations within the monomer ensemble. Increased fibril-native intrachain contact probability lowers the entropic cost of productive intermolecular alignment during early oligomerization, thereby biasing the nucleation propensity. Thus, our conclusions concern the modulation of aggregation-competent conformations rather than a direct simulation of multimeric assembly.

Importantly, these residue-specific effects also provide a mechanistic context for the previously observed Spm-induced phase separation of Tau and αS.^34^ While our prior work demonstrated the formation of highly dynamic liquid-like condensates, the present analysis suggests that Spm can simultaneously suppress fibril-like interactions in Tau while enhancing aggregation-associated contacts in αS. This decoupling of condensation behavior from aggregation propensity highlights that LLPS alone does not uniquely determine disease-relevant outcomes. Previous studies have reported that aggregation of both Tau^80^ and αS^81^ can emerge from their phase-separated states under certain conditions, whereas divergent salt dependence in αS^56^ and RNA-dependent regulation of FUS^82^ demonstrate that this coupling is not universal. In this context, our results show that Spm-driven residue-level reweighting of fibril-native contacts biases the outcome of intermolecular encounters within condensates. Together, these findings underscore that residue-level interaction patterns govern whether condensates mature toward or away from aggregates under different conditions.

To further elucidate the structural basis of these divergent behaviors under Spm, we analyzed both global and local structural changes. The net attraction or repulsion observed in the radial distribution functions could be fully explained by the overall net charge of each protein chain. However, the radius of gyration values did not follow a simple monotonic trend with Spm concentration, indicating specific Spm–IDP interactions beyond global charge effects. All simulations were performed at physiological ionic strength, and although both salt concentration and absolute Spm levels modulate electrostatic screening and interaction strength, the residue-specific interaction patterns and qualitative aggregation trends remain consistent across the explored concentration regimes and are supported by ThT measurements at lower, physiologically relevant protein–Spm ratios.

Secondary structure analysis further revealed notable changes in regions known to be important for fibril formation. Residue-specific binding analysis demonstrated preferential interactions of Spm with specific segments in each IDP, with clear structural consequences: in K18, the region with the strongest Spm binding coincided with major contact disruptions in the aggregation core; in αS, Spm primarily bound to the CTD, reducing CTD–NAC interactions and altering NAC accessibility; and in Aβ40, binding near residues 23–24 was accompanied by local compaction of adjacent regions. Thus, Spm exerts divergent protein-specific influences on aggregation.

The lack of well-defined binding pockets in IDPs presents a major obstacle to traditional drug design. As such, exploring alternative strategies, such as modulating cellular polyamine levels, may offer novel therapeutic avenues for controlling IDP aggregation. Our findings suggest that regulating spermine concentration could potentially modulate IDP aggregation and more broadly reshape the conformational landscapes of disease-associated IDPs. Importantly, all-atom simulations of monomeric proteins prove valuable in revealing residue-specific interactions that underlie these effects, offering mechanistic insight into how small molecules such as polyamines modulate complex aggregation behaviors. Future work should extend these approaches to more aggregation-prone variants and cellular contexts to evaluate the therapeutic potential of targeting polyamine–protein interactions.

## Methods

### All-Atom MD Simulation

The all-atom MD simulations were performed using GROMACS version 2023.3 software package^83,84^ for all the systems. We have used AMBER ff99SBws^85^ force field for the proteins along with TIP4P/2005 water model,^86^ and general AMBER force field^87^ (GAFF) for Spm. Initial conformations for all systems were generated using the I-TASSER webserver.^88^ To obtain disordered starting conformations, each model was subjected to a 20 ns NVT simulated-annealing protocol in which the temperature was gradually increased from 300 to 500 K and subsequently cooled back to 300 K in the absence of solvent and ions. The resulting disordered structures were then solvated with water and ions prior to equilibration and production simulations. The structures for all three systems were inserted in a triclinic dodecahedron simulation box. Following this, the required number of Spm molecules was added. The simulation boxes are filled with water molecules, and the required numbers of sodium and chloride ions are added to obtain ∼150 mM NaCl concentration along with ions to neutralize the systems. The Antechamber module^89^ has been used to generate the GAFF parameters for Spm. Atomic partial charges were derived using the restrained electrostatic potential (RESP) fitting procedure^90^ based on electrostatic potentials computed at the HF/6–31G* level of theory. The systems were first energy-minimized using the steepest descent method. This was followed by a 1 ns equilibration simulation under constant pressure and temperature using Berendsen pressure coupling^91^ and V-rescale temperature coupling.^92^ The same coupling constant of 1 ps has been used for both temperature and pressure coupling during the equilibration. For the K18 systems, the simulations were run at 283 K in accordance with the NMR experiments carried out for this system. For αS and Aβ40, the simulations were carried out at 288 and 278 K, respectively, to make the simulation conditions consistent with previously reported NMR experiments on these systems.^31,93^ Equilibrated structures were used for production simulation under constant pressure and temperature conditions. Parrinello–Rahman barostat^94^ has been used along with the V-rescale thermostat for these simulations with a coupling constant of 1 ps for both barostat and thermostat. Electrostatic interactions were treated using PME electrostatics^95^ with a 1.2 nm cutoff. The van der Waals cutoff was set to 1.2 nm. The initial 500 ns has been discarded from K18 and Aβ40 simulations, while for αS simulations, the initial 200 ns has been discarded. We calculated the percentage of trajectory frames in which the minimum distance to the periodic image was less than 0.3 nm and found it to be negligible (Table S1). All subsequent analyses were performed after excluding these frames.

To evaluate whether the production simulations sufficiently sampled intrachain contact dynamics, we quantified contact relaxation times for all residue pairs. Pairwise heavy-atom distances were converted into binary contact trajectories using a twin-cutoff scheme, in which a contact was defined as formed when the distance was <0.45 nm and considered broken when it exceeded 0.6 nm. Time-correlation analysis was then performed on these contact trajectories, and characteristic contact relaxation times were obtained by fitting the exponential decay of the contact autocorrelation. The distributions of contact relaxation times for all three systems are shown in Figure S17. Most contact lifetimes are on the order of tens of nanoseconds, substantially shorter than the total simulation lengths (2–5 μs; Table S1). These results indicate that intrachain contacts form and dissociate multiple times within each trajectory, supporting adequate sampling of contact dynamics in the simulations.

### Simulation Analyses

The radial distribution functions, *R*_g_ values, and secondary structure assignments were obtained using GROMACS for all the systems. The *R*_g_ values are given in Table S1, and the errors were estimated by using a block averaging method with four blocks. From these assignments, the probability of each residue in coil, helix, or sheet conformation has been determined. For calculating the number of Spm around each protein residue, the number of Spm that comes in contact with each residue has been calculated. For this purpose, a contact has been considered between Spm and residues if any of their non-hydrogen atoms come within a distance of 0.45 nm of each other. The same condition has been used for obtaining the intraresidue contact maps for all the systems. In all the cases, residues that are separated by at least three other residues have been considered. For Spm-mediated contacts, the distance cutoff chosen between non-hydrogen atoms of Spm and protein residues is 0.6 nm. In this case also, we have taken only those residues that are separated by at least three other residues.

To characterize the dynamic association of spermine with protein residues, additional 150 ns simulations were performed at 300 K. Simulations were carried out at a 1:50 protein:Spm ratio for K18 and αS, and at a 1:5 ratio for Aβ40. Trajectories were saved every 0.25 ps, and the final 100 ns were used for residence-time analysis. Two complementary residence-time metrics were computed. The local residue residence time τ_residue_ was defined as the time for a spermine molecule initially within 0.45 nm of a given residue to move beyond 0.6 nm from that residue. The protein-level residence time τ_protein_ was defined as the time required for the spermine molecule initially within 0.45 nm of a given residue to move beyond 0.6 nm from all protein residues, thereby quantifying complete dissociation from the protein surface. The exchange rate was computed as the number of dissociation events at each residue divided by the total analyzed trajectory time (events per nanosecond), enabling discrimination between long-lived binding and frequent transient exchange of spermine molecules.

### Protein Purification

Full-length human Tau (441 amino acids, UniProt P10636–8) and K18 variant (residues 244–372) were expressed in *E. coli* BL21 (DE3) and purified as previously described.^96^ Expression was performed at 37 °C in LB medium with ampicillin (100 mg/L). IPTG (0.4 mM final concentration) was added at OD600 0.6–0.8, followed by 2 h of cultivation. Cells were harvested and stored at −20 °C. Cell pellets were resuspended in 50 mM NaPi and 2.5 mM EDTA, pH 6.2, with protease inhibitors. Soluble extracts were obtained by sonication and centrifugation. Supernatants were heated at 75 °C for 15 min and then centrifuged. Proteins were purified using Hi-Trap SP FF cation exchange chromatography with a NaCl gradient elution (50 mM NaPi, 2.5 mM EDTA, 500 mM NaCl, pH 6.2). Purified proteins were verified by 12% SDS-PAGE, dialyzed against 20 mM ammonium bicarbonate, and lyophilized. Concentrations were determined by UV absorption at 280 nm (extinction coefficients: Tau 7450 M^–1^ cm^–1^, K18 1490 M^–1^ cm^–1^).

### ThT Kinetics

20 μM Tau or K18 was incubated with varying spermine ratios in the presence of two glass beads (1.0 mm diameter, Sigma-Aldrich) and 50 μM ThT in 25 mM HEPES (pH 7.4). Samples were prepared on ice, and 20 μL aliquots were dispensed into 384-well black/clear-bottom microplates (ThermoFisher, catalog no. 242764) and sealed using aluminum adhesive foil (neoLab). Aggregation was monitored using a PHERAstar FSX microplate reader (BMG LABTECH, Germany) at 37 °C with 300 rpm shaking. ThT fluorescence was measured every 7 min (excitation 430 nm, emission 480 nm). Each condition was measured in three repeats. The mean and standard error of the mean were calculated for subsequent analyses. The ThT fluorescence time-course data were fitted to a sigmoidal growth model using the logistic function:

$$F\left(t\right)=F_{0}+\frac{F_{max}-F_{0}}{1+\exp\left[-k\left(t-t_{1/2}\right)\right]}$$

where *F*(*t*) is the fluorescence intensity at time *t*. *F*_0_ represents the initial baseline fluorescence prior to aggregation, and *F*_max_ denotes the final plateau fluorescence after aggregation reaches completion. The parameter *k* is the apparent rate constant describing the steepness of the growth phase, with larger values indicating a faster transition. The term *t*_1/2_ corresponds to the half-time of the reaction, defined as the time at which fluorescence reaches halfway between *F*_0_ and *F*_max_. Lag time was calculated as *t*_1/2_ – 2/*k*, consistent with the standard tangent-intersection method used for sigmoidal ThT kinetics. The uncertainty in the fitted parameters was estimated using a bootstrapping procedure.

## Supplementary Material



## Notes

Supporting tables detailing simulation parameters, PDB structures used, and fitting parameters for ThT kinetic data, along with supporting figures presenting additional simulation analyses (PDF)
