# Supplementary material for: Residue-Specific Modulation of Aggregation-Associated Interactions by Spermine in Tau, α‑Synuclein, and Aβ40
Source: JACS Au. 2026 Mar 12;6(3):2040–54. doi: 10.1021/jacsau.6c00126 (PMC13014203; doi:10.1021/jacsau.6c00126)
Supplement: Supplementary file 1 [file au6c00126_si_001.pdf]

SUPPORTING INFORMATION
For

Residue-Specific Modulation of Aggregation-Associated Interactions by Spermine in Tau, α-Synuclein, and Aβ40

Debasis Saha[1*], Xun Sun[2], Wangfei Yang[1], Jinghui Luo[2] and Wenwei Zheng[1,3*]

[1]College of Integrative Sciences and Arts, Arizona State University, Mesa, AZ, 85212, USA
[2]Center for Life Sciences, Paul Scherrer Institute, Forschungsstrasse 111, 5232 Villigen PSI, Switzerland
[3]Center for Biological Physics, Arizona State University, Tempe, AZ, 85281, USA

[*]Email: dsaha3@asu.edu, wenweizheng@asu.edu

Supporting Tables

Table S1. Simulation details for all the systems along with their radius of gyration (R_g) and Flory scaling exponent (v) values.

System	Protein-Spm ratio	Box size (nm)	No. of atoms	Simulation lengths (μs)	% of Frames (Min. dist. ≥ 0.3 nm)	R_g (nm)	ν
K18	1:0	12.08 × 12.08 × 8.54	166881	5	95.6	3.61	0.594
	1:10	12.09 × 12.09 × 8.55	168233	5	97.6	3.79	0.600
	1:20	12.07 × 12.07 × 8.53	166553	5	94.1	4.08	0.621
	1:50	12.07 × 12.07 × 8.53	167513	5	95.9	3.75	0.598
	1:100	12.12 × 12.12 × 8.57	169113	5	99.4	3.50	0.589
αS	1:0	14.89 × 14.89 × 10.53	312963	2	100.0	3.44	0.566
	1:20	14.97 × 14.97 × 10.59	316691	2	100.0	3.37	0.577
	1:50	14.93 × 14.93 × 10.56	314563	2	99.4	3.83	0.590
	1:100	14.97 × 14.97 × 10.59	317387	2	100.0	3.61	0.573
Aβ40	1:0	7.17 × 7.17 × 5.07	34911	5	99.8	1.74	0.580
	1:2	7.17 × 7.17 × 5.07	34931	5	96.6	1.77	0.594
	1:5	7.17 × 7.17 × 5.07	34979	5	99.8	1.68	0.570

Table S2. List of PDBs for Tau fibrils belonging to different clusters.

Cluster Id	PDB file name
**Cluster 1**	7QJV[1], 7QL4[1] , 7QKI[1], 7QKK[1], 5O3L[2], 5O3T[2], 5O3O[2], 6HRE[3], 7NRS[4], 7NRX[4], 7NRQ[4], 7NRV[4], 7NRT[4]
**Cluster 2**	7QJW[1], 7QJZ[1], 7QK5[1], 7QKU[1], 7QKV[1], 7QKX[1], 7QL0[1], 7QL1[1], 7QKG[1], 7QK6[1], 7QKW[1], 6NWP[5], 7QK1 (PF1*)[1]
**Cluster 3**	7QL3[1], 7QJY[1], 7QKJ[1], 7QK2[1], 7QK1 (PF2*)[1]
**Cluster 4**	7QKL[1], 7R4T[1], 7QJX[1]
**Cluster 5**	6TJX[6], 6TJO[6]
**Cluster 6**	7R5H[1]
**Cluster 7**	7QKF[1]
**Cluster 8**	7QL2[1]
**Cluster 9**	7QKZ[1]
**Cluster 10**	7QK3[1]
**Cluster 11**	7QKH[1]
**Cluster 12**	7QKY[1]
**Cluster 13**	7QKM[1]
**Cluster 14**	6GX5[7]

*PF1 and PF2 indicate two conformationally different protofilaments belonging to the same PDB entry.

Table S3. List of PDBs for αS fibrils belonging to different clusters.

Cluster Id	PDB file name
Cluster 1	6SST[8], 6SSX[8]
Cluster 2	6XYP[9], 6XYQ[9]
Cluster 3	6H6B[10]
Cluster 4	8A9L[11]
Cluster 5	6XYO[9]

Table S4. Phenomenological and fitting parameters for amyloid aggregation for K18, derived from sigmoidal curve fitting to Thioflavin T (ThT) fluorescence kinetics data.

System	Spm ratio	F_0 (a.u.)	F_{Max} (a.u.)	k (h^{-1})	$t_{1/2}$ (h)	t_{lag} (h)
K18 (res. 244-372)	1:0	200.85 ± 1.71	802.20 ± 3.57	1.62 ± 0.30	18.45 ± 0.35	17.19 ± 0.37
	1:1	151.50 ± 1.16	693.53 ± 6.57	0.34 ± 0.01	30.78 ± 0.11	24.91 ± 0.15
	1:5	101.64 ± 0.94	385.89 ± 3.85	0.15 ± 0.01	39.64 ± 0.30	26.47 ± 0.34

Supporting Figures

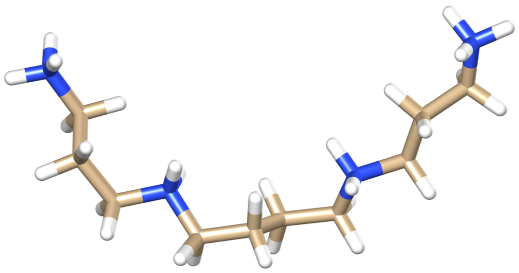

Figure S1. The structure of spermine (Spm) with a net charge of +4.

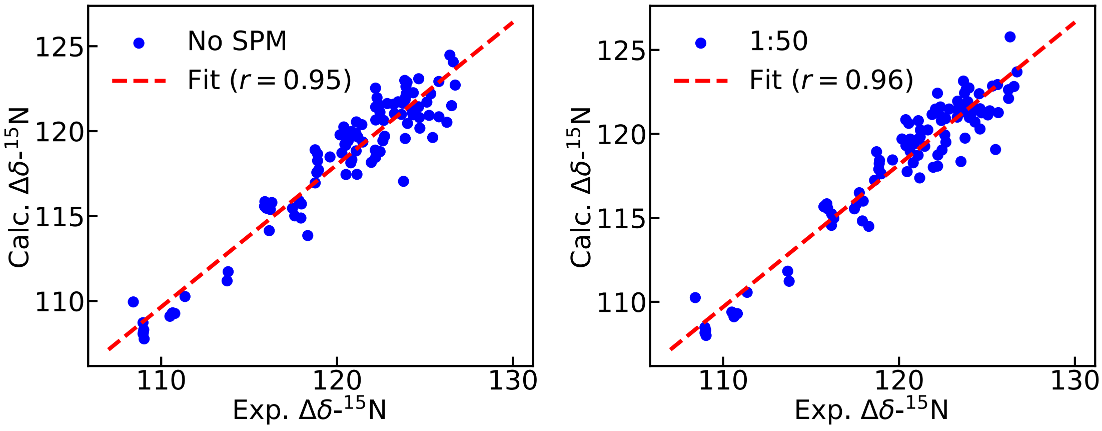

Figure S2. Comparison between experimental and calculated NMR chemical shifts. Calculated vs experimental NMR chemical shifts for ^{15}N atoms of K18 system in the absence (left) and presence of Spm (right) at 1:50 protein-Spm ratio. The red dashed line shows the linear fitting of the data with Pearson correlation coefficient (r) values given in the plots.

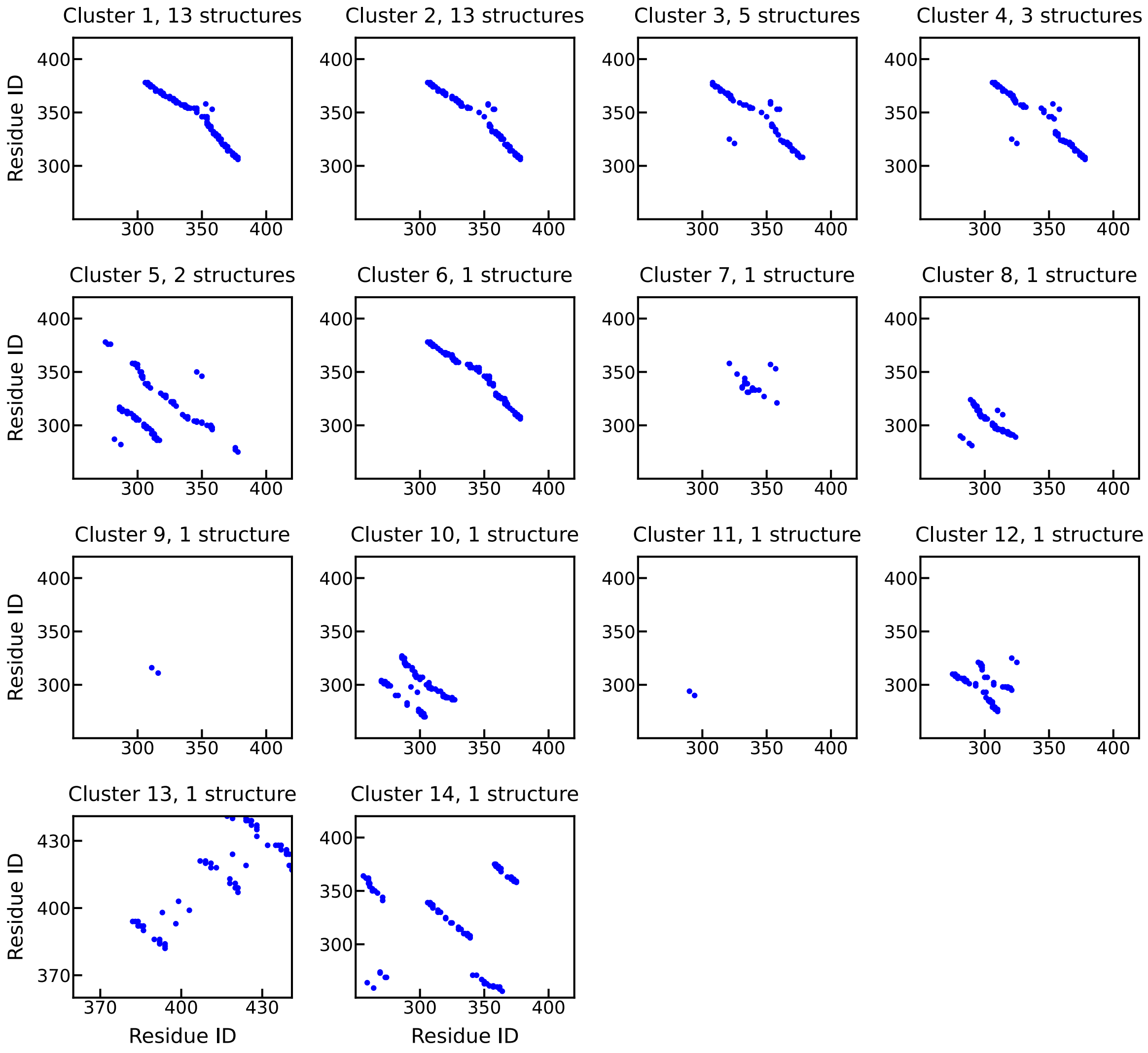

Figure S3. Contact map of different Tau fibril structures. Intraresidue contacts maps of the 14 clusters obtained from Tau fibrils. The number of structures belonging to each cluster is mentioned at the top of each contact map.

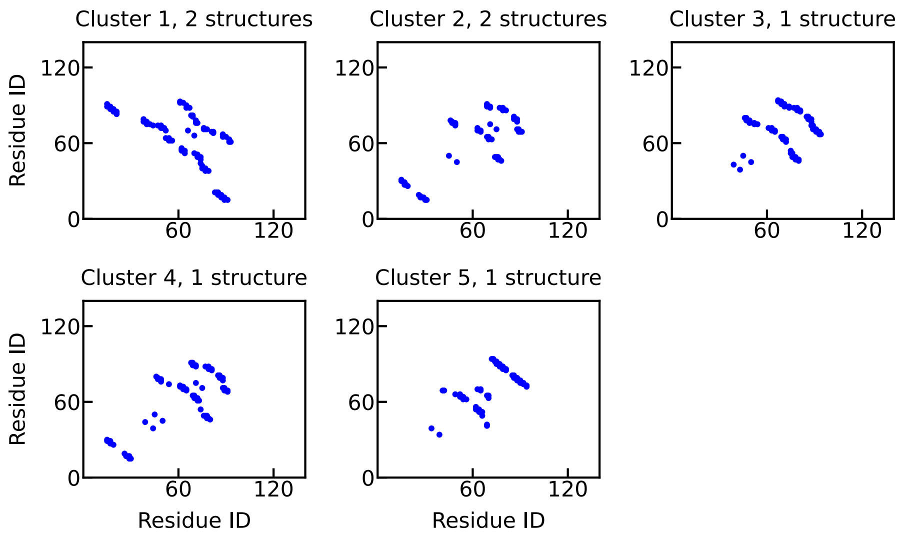

Figure S4. Contact map of different αS fibril structures. Intraresidue contact maps of the five clusters obtained from αS fibrils. The number of structures belonging to each cluster is mentioned at the top of each contact map.

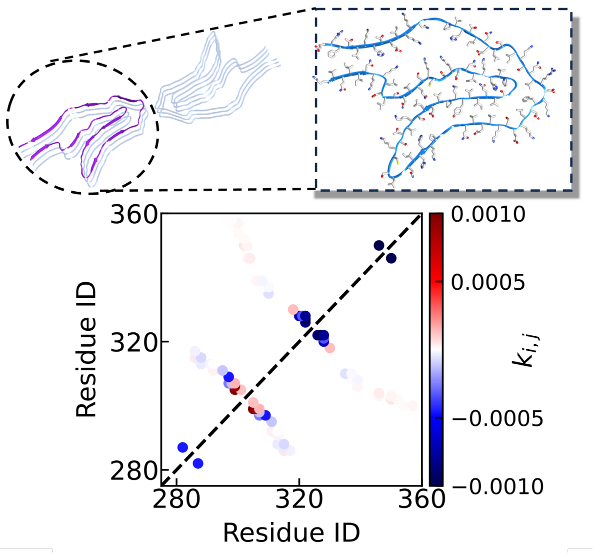

Figure S5. Structure and contact modulation map for Tau fibril associated with Corticobasal Degeneration (CBD). The fibril structure of CBD related Tau fibril (PDB ID: 6TJX) with one monomer highlighted in purple and shown in the zoomed in structure on the right. The corresponding contact modulation map showing modulation coefficients $k_{i,j}$ for contact pairs present in the fibril structure.

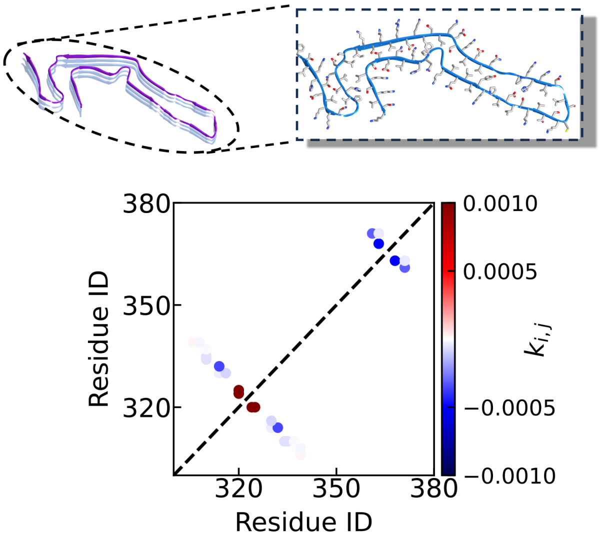

Figure S6. Structure and contact modulation map for Tau fibril associated with Pick's disease. The fibril structure of Pick's disease related Tau fibril (PDB ID: 6GX5) with one monomer highlighted in purple and shown in the zoomed in structure on the right. The corresponding contact modulation map showing modulation coefficients $k_{i,j}$ for contact pairs present in the fibril structure.

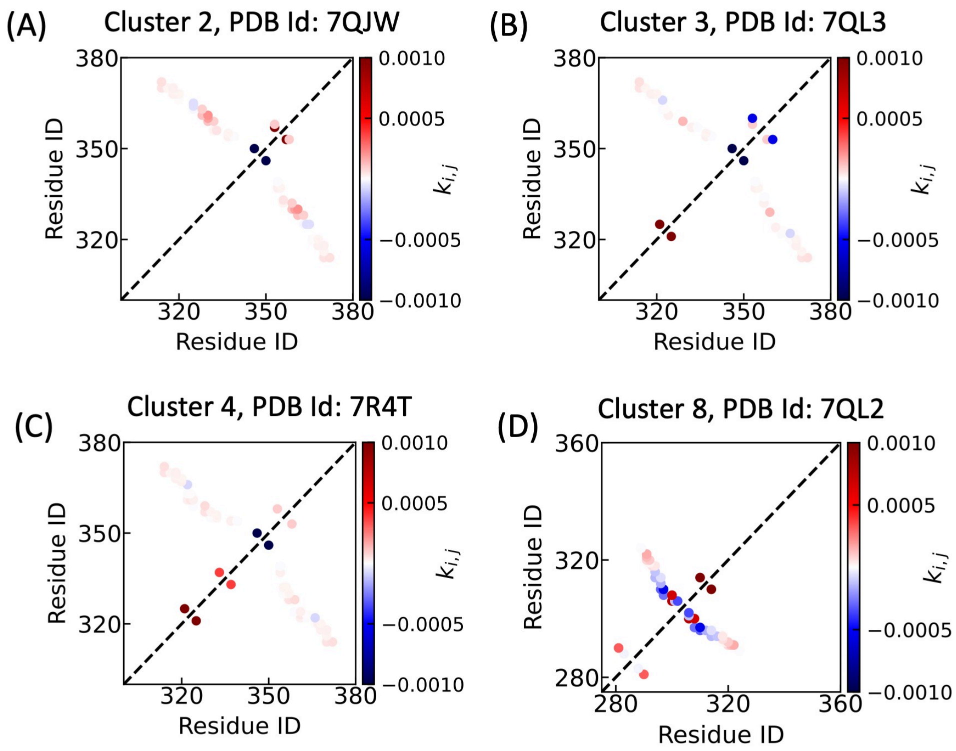

Figure S7. Contact modulation maps for Tau fibril structures belonging to different structural clusters. The maps show the modulation coefficients $k_{i,j}$ for residue pairs that form native contacts in the corresponding fibril structures, calculated using the protocol described in Fig. 1. The PDB identifier and cluster number are indicated at the top of each contact map.

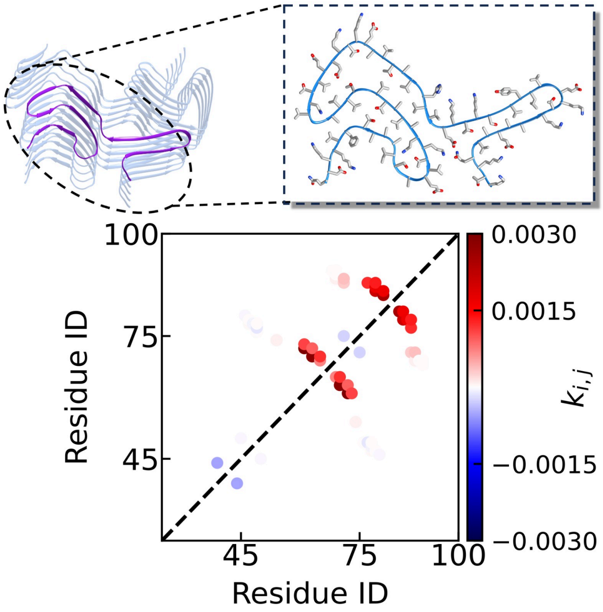

Figure S8. Structure and contact modulation map for αS fibril associated with Multiple System Atrophy (MSA). The fibril structure of MSA related αS fibril with one monomer highlighted in purple and shown in the zoomed in structure on the right. The corresponding contact modulation map showing modulation coefficients $k_{i,j}$ for contact pairs present in the fibril structure.

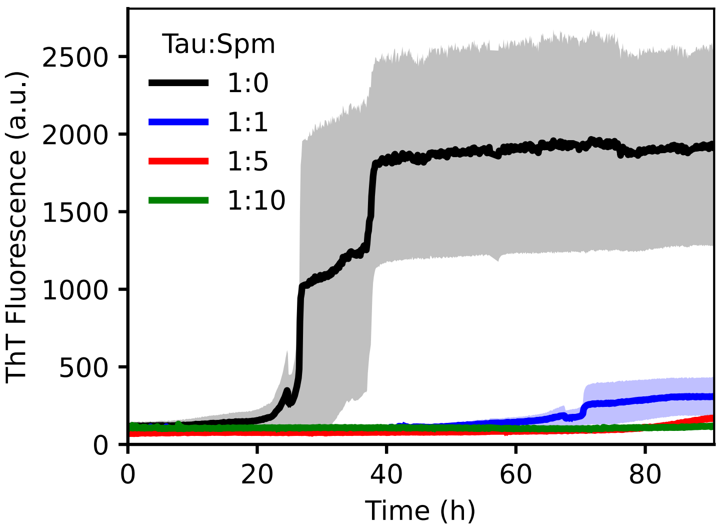

Figure S9. ThT Fluorescence of full-length Tau. For each Tau-Spm ratio, the data shows the average plot along with their standard errors of the mean.

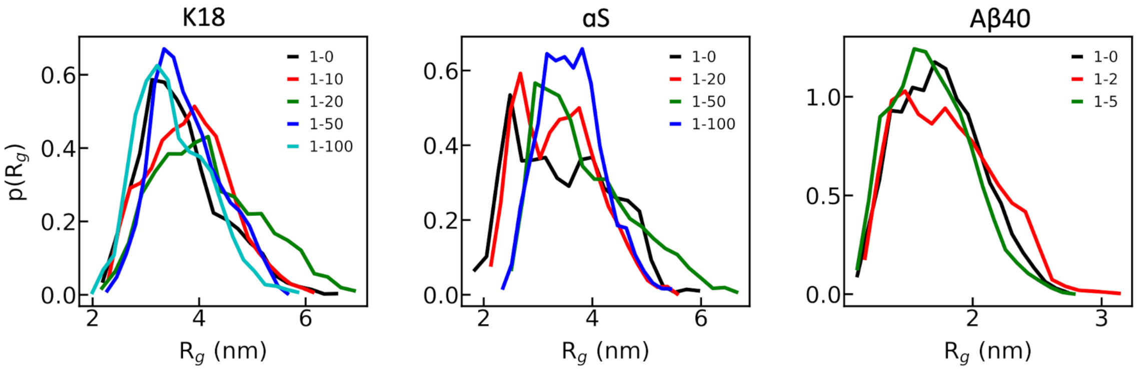

Figure S10. Distribution of R_g-s. The distribution of R_g-s with and without Spm for the three systems.

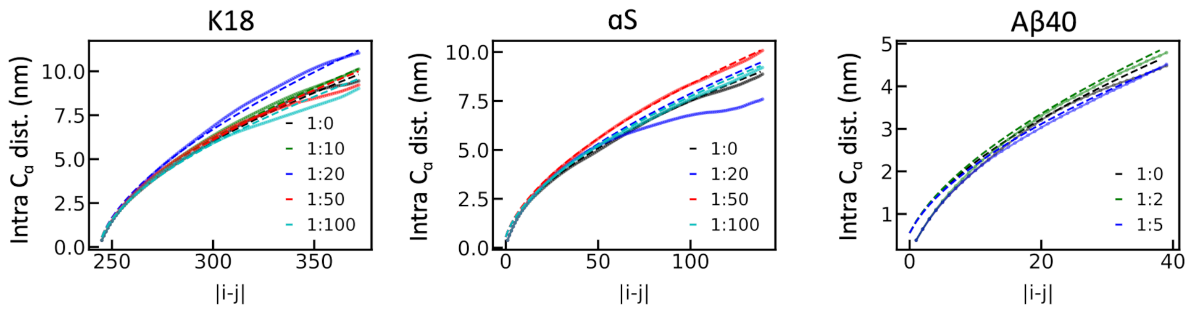

Figure S11. Scaling exponent calculations. The intrachain distances as a function of the sequence separation (solid lines) and their fits (dashed lines) for the three systems with and without Spm.

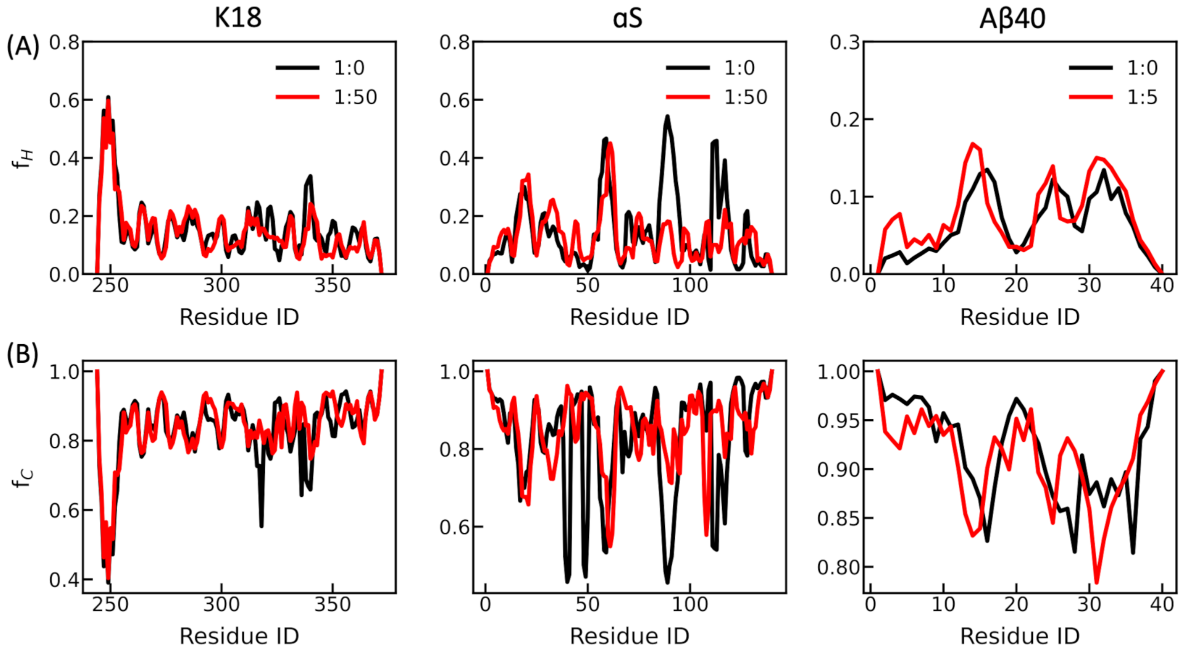

Figure S12. Secondary structure analysis for the three systems. (A) The fraction of helical conformations, f_H, and (B) coil conformations, f_C for K18 (left panel), αS (middle panel) and Aβ40 (right panel).

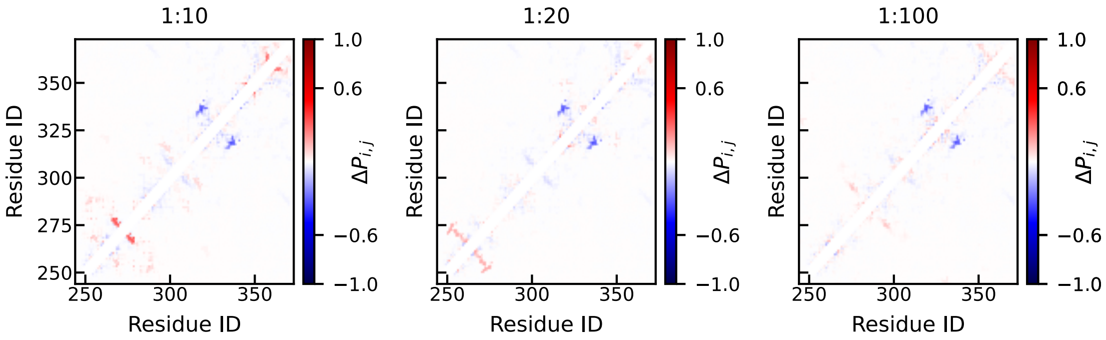

Figure S13. Changes in contact maps for K18 system at various Spm ratios. The changes represent the intraresidue contact probability changes between simulations with and without Spm.

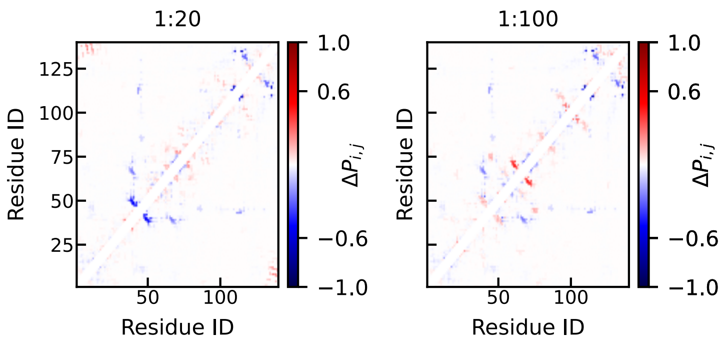

Figure S14. Changes in contact maps for αS system at various Spm ratios. The changes represent the intraresidue contact probability changes between simulations with and without Spm.

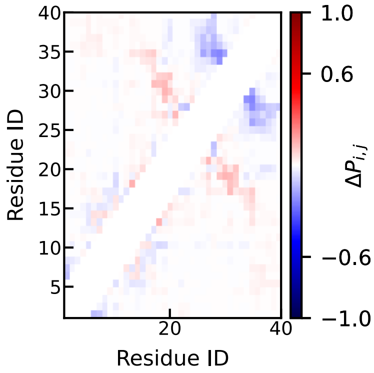

Figure S15. Changes in contact maps for Aβ40 system at 1:2 Spm ratio. The change represent the intraresidue contact probability changes between simulations with and without Spm.

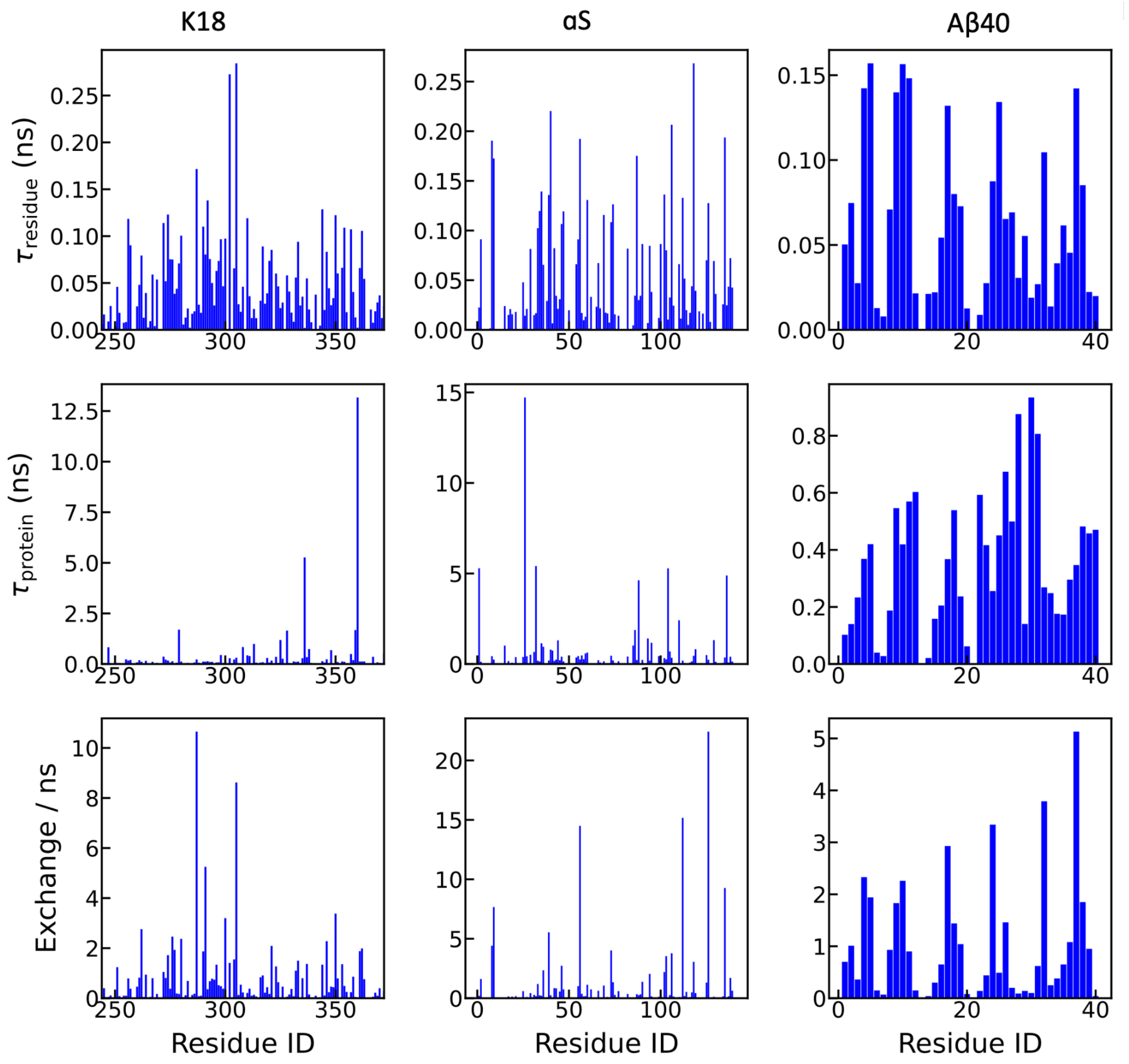

Figure S16. Residue-level residence times and exchange dynamics of Spm. Two complementary residence-time measures and the corresponding exchange rates are shown for K18 (left), αS (middle), and Aβ40 (right). The top panels present the local residence time ($\tau_{residue}$), defined as the average duration for which a Spm molecule, initially within 0.45 nm of a given residue, remains associated before moving beyond 0.6 nm from that same residue. The middle panels show the protein-level residence time ($\tau_{protein}$), defined as the average time required for a Spm molecule initially within 0.45 nm of a given residue to move beyond 0.6 nm from all protein residues, thereby quantifying complete dissociation from protein surface. The bottom panels display the corresponding exchange rate (events per ns), calculated from the total number of Spm dissociation events at a given residue divided by the total analyzed trajectory time, thereby quantifying how frequently Spm molecules associate and dissociate at each residue.

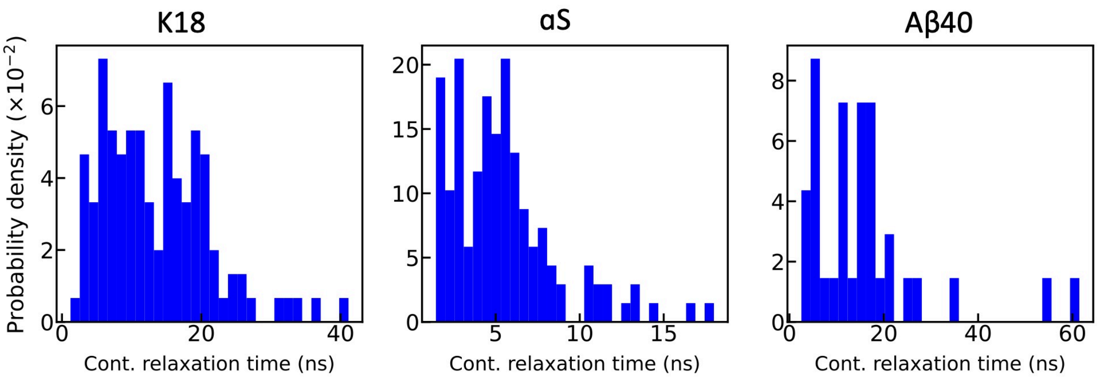

Figure S17. Distribution of contact relaxation times for residue pairs. Characteristic relaxation times obtained from contact time correlation analysis are shown for K18 (left), αS (middle), and Aβ40 (right). For K18 and αS, the 1:50 protein:Spm simulations were analyzed, whereas for Aβ40 the 1:5 system was used.

Supporting References

(1) Lövestam, S.; Koh, F. A.; van Knippenberg, B.; Kotecha, A.; Murzin, A. G.; Goedert, M.; Scheres, S. H. W. Assembly of Recombinant Tau into Filaments Identical to Those of Alzheimer's Disease and Chronic Traumatic Encephalopathy. *Elife* **2022**, *11*. https://doi.org/10.7554/ELIFE.76494.

(2) Fitzpatrick, A. W. P.; Falcon, B.; He, S.; Murzin, A. G.; Murshudov, G.; Garringer, H. J.; Crowther, R. A.; Ghetti, B.; Goedert, M.; Scheres, S. H. W. Cryo-EM Structures of Tau Filaments from Alzheimer's Disease. *Nature 2017 547:7662* **2017**, *547* (7662), 185–190. https://doi.org/10.1038/nature23002.

(3) Falcon, B.; Zhang, W.; Schweighauser, M.; Murzin, A. G.; Vidal, R.; Garringer, H. J.; Ghetti, B.; Scheres, S. H. W.; Goedert, M. Tau Filaments from Multiple Cases of Sporadic and Inherited Alzheimer's Disease Adopt a Common Fold. *Acta Neuropathol* **2018**, *136* (5), 699–708. https://doi.org/10.1007/s00401-018-1914-z.

(4) Shi, Y.; Murzin, A. G.; Falcon, B.; Epstein, A.; Machin, J.; Tempest, P.; Newell, K. L.; Vidal, R.; Garringer, H. J.; Sahara, N.; Higuchi, M.; Ghetti, B.; Jang, M.-K.; Scheres, S. H. W.; Goedert, M. Cryo-EM Structures of Tau Filaments from Alzheimer's Disease with PET Ligand APN-1607. *Acta Neuropathol* **2021**, *141* (5), 697–708. https://doi.org/10.1007/s00401-021-02294-3.

(5) Falcon, B.; Zivanov, J.; Zhang, W.; Murzin, A. G.; Garringer, H. J.; Vidal, R.; Crowther, R. A.; Newell, K. L.; Ghetti, B.; Goedert, M.; Scheres, S. H. W. Novel Tau Filament Fold in Chronic Traumatic Encephalopathy Encloses Hydrophobic Molecules. *Nature 2019 568:7752* **2019**, *568* (7752), 420–423. https://doi.org/10.1038/s41586-019-1026-5.

(6) Zhang, W.; Tarutani, A.; Newell, K. L.; Murzin, A. G.; Matsubara, T.; Falcon, B.; Vidal, R.; Garringer, H. J.; Shi, Y.; Ikeuchi, T.; Murayama, S.; Ghetti, B.; Hasegawa, M.; Goedert, M.; Scheres, S. H. W. Novel Tau Filament Fold in Corticobasal Degeneration. *Nature 2020 580:7802* **2020**, *580* (7802), 283–287. https://doi.org/10.1038/s41586-020-2043-0.

(7) Falcon, B.; Zhang, W.; Murzin, A. G.; Murshudov, G.; Garringer, H. J.; Vidal, R.; Crowther, R. A.; Ghetti, B.; Scheres, S. H. W.; Goedert, M. Structures of Filaments from Pick's Disease Reveal a Novel Tau Protein Fold. *Nature 2018 561:7721* **2018**, *561* (7721), 137–140. https://doi.org/10.1038/s41586-018-0454-y.

(8) Guerrero-Ferreira, R.; Taylor, N. M.; Arteni, A.-A.; Kumari, P.; Mona, D.; Ringler, P.; Britschgi, M.; Lauer, M. E.; Makky, A.; Verasdonck, J.; Riek, R.; Melki, R.; Meier, B. H.; Böckmann, A.; Bousset, L.; Stahlberg, H. Two New Polymorphic Structures of Human Full-Length Alpha-Synuclein Fibrils Solved by Cryo-Electron Microscopy. *Elife* **2019**, *8*. https://doi.org/10.7554/eLife.48907.

(9) Schweighauser, M.; Shi, Y.; Tarutani, A.; Kametani, F.; Murzin, A. G.; Ghetti, B.; Matsubara, T.; Tomita, T.; Ando, T.; Hasegawa, K.; Murayama, S.; Yoshida, M.; Hasegawa, M.; Scheres, S. H. W.; Goedert, M. Structures of α-Synuclein Filaments from Multiple System Atrophy. *Nature 2020 585:7825* **2020**, *585* (7825), 464–469. https://doi.org/10.1038/s41586-020-2317-6.

(10) Guerrero-Ferreira, R.; Taylor, N. M.; Mona, D.; Ringler, P.; Lauer, M. E.; Riek, R.; Britschgi, M.; Stahlberg, H. Cryo-EM Structure of Alpha-Synuclein Fibrils. *Elife* **2018**, *7*. https://doi.org/10.7554/eLife.36402.

(11) Yang, Y.; Shi, Y.; Schweighauser, M.; Zhang, X.; Kotecha, A.; Murzin, A. G.; Garringer, H. J.; Cullinane, P. W.; Saito, Y.; Foroud, T.; Warner, T. T.; Hasegawa, K.; Vidal, R.; Murayama, S.; Revesz, T.; Ghetti, B.; Hasegawa, M.; Lashley, T.; Scheres, S. H. W.; Goedert, M. Structures of α-Synuclein Filaments from Human Brains with

Lewy Pathology. *Nature 2022 610:7933* **2022**, *610* (7933), 791–795. https://doi.org/10.1038/s41586-022-05319-3.
